# Sepsis: Epidemiology, Pathophysiology, and Management

**DOI:** 10.1002/mco2.71079

**Published:** 2026-10-27

**Authors:** Guang‐Tao Pan, Yu Deng, Lin‐Rui Ma, Yu‐Tong Li, Yu Qian, Yi‐Hua Ma, Hai‐Wei Rao, Yi‐Zhun Zhu, Pei‐Yu Yan

**Affiliations:** ^1^ Yancheng Hospital of Traditional Chinese Medicine Affiliated to Nanjing University of Chinese Medicine (Yancheng Hospital of Traditional Chinese Medicine) Yancheng Jiangsu China; ^2^ Faculty of Chinese Medicine Macau University of Science and Technology Macau SAR China; ^3^ School of Pharmacy Macau University of Science and Technology Macau SAR China; ^4^ Laboratory of Drug Discovery from Natural Resources and Industrialization Macau University of Science and Technology Macau SAR China; ^5^ Faculty of Chinese Medicine State Key Laboratory of Mechanism and Quality of Chinese Medicine Macau University of Science and Technology Macau SAR China; ^6^ Zhuhai MUST Science and Technology Research Institute Zhuhai Guangdong China

**Keywords:** epidemiology, management, natural compounds, pathophysiology, precision immunotherapy, programmed cell death, sepsis

## Abstract

Sepsis remains a major cause of global morbidity and mortality despite advances in recognition, antimicrobial therapy, and organ support, reflecting its biological heterogeneity and the absence of broadly effective host‐directed treatments. Here, we synthesize recent evidence across the three domains that define contemporary sepsis: epidemiology, pathophysiology, and management. Global and regional data indicate a persistently high but uneven burden, shaped by differences in case definitions, population structure, healthcare resources, and ascertainment. Mechanistically, sepsis is best viewed as an interconnected systems disorder in which hyperinflammation and immunoparalysis coexist with oxidative stress, endothelial and coagulation injury, immunometabolic dysfunction, gut dysbiosis, mitochondrial damage, and dysregulated programmed cell death, collectively driving organ dysfunction. Current management remains centered on timely antimicrobials, source control, individualized fluid and vasopressor therapy, corticosteroids, and organ support, while biomarker‐guided immunotherapy and other host‐directed strategies remain investigational. Recent randomized trials have mainly refined how established treatments are delivered rather than identified a universally effective new therapy. We also evaluate preclinical evidence for natural compounds targeting inflammatory and cell‐death networks and highlight persistent methodological, pharmacokinetic, and translational barriers. Integrating rigorous phenotyping, mechanistic biomarkers, and guideline‐based care may provide a more realistic path toward precision sepsis therapy.

## Graphical abstract

Sepsis remains a major global health burden characterized by substantial geographical heterogeneity and a considerable disease burden in China. Its pathophysiology reflects an interconnected network involving excessive inflammation, oxidative stress, immune imbalance and immunoparalysis, mitochondrial damage, endothelial injury, gut dysbiosis, immunometabolic dysfunction, and dysregulated programmed cell death (PCD), which collectively contribute to target‐organ injury and multiple‐organ dysfunction syndrome (MODS). Contemporary management is centered on timely antimicrobial therapy and source control, hemodynamic stabilization, organ‐supportive care, and individualized immune‐directed and adjunctive therapies. Precision medicine based on clinical phenotyping and biomarker‐guided stratification represents an emerging direction, whereas PCD‐targeting natural compounds remain potential therapeutic candidates requiring further mechanistic, pharmacokinetic, and clinical validation.

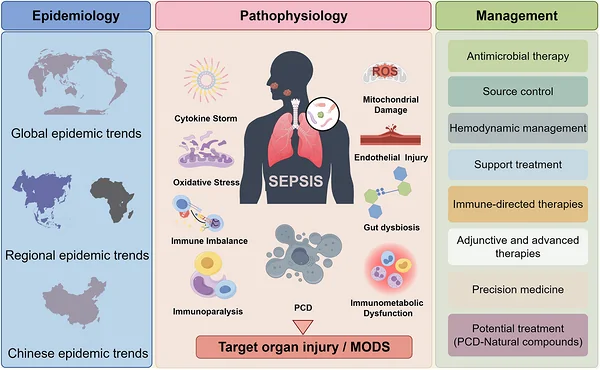

## 1 Introduction

Sepsis is a life‐threatening syndrome characterized by organ dysfunction caused by a dysregulated host response to infection and remains a major challenge in critical care worldwide [1, 2]. The Global Burden of Disease Study 2021 estimated approximately 166 million incident sepsis cases and 21.4 million sepsis‐related deaths worldwide in 2021 [3]. In China, the standardized incidence of hospitalized sepsis increased from 328.25 per 100,000 population in 2017 to 421.85 per 100,000 population in 2019, with substantial geographic variation [4]. These estimates highlight the continuing global burden of sepsis while also emphasizing the importance of considering differences in case definitions, data sources, healthcare access, and regional ascertainment. Current clinical management is based on early recognition, appropriate antimicrobial therapy, source control, hemodynamic stabilization, organ support, and prevention of secondary complications, as outlined in the 2026 Surviving Sepsis Campaign guidelines [5].

Despite advances in early diagnosis, antimicrobial treatment, and intensive care, outcomes remain highly variable and long‐term disability remains common. Sepsis is not adequately described by a simple transition from early hyperinflammation to later immunosuppression. Instead, pathogen‐associated molecular patterns (PAMPs) and damage‐associated molecular patterns (DAMPs) activate interconnected inflammatory, endothelial, coagulation, mitochondrial, metabolic, and tissue‐injury responses [6]. Hyperinflammatory and immunosuppressive programs may coexist in different tissues and at different time points. Their relative contribution is influenced by the causative pathogen, infection source, host age, comorbidities, treatment timing, and affected organ compartment. This biological heterogeneity helps explain why interventions that are effective in selected patients may not produce consistent benefits when applied to unselected populations.

Within this integrated pathophysiological network, programmed cell death (PCD) represents an important mechanistic layer. Established regulated cell‐death mechanisms were defined by the Nomenclature Committee on Cell Death framework [7], whereas newer concepts, including PANoptosis and cuproptosis, have subsequently been investigated in sepsis [8, 9]. Autophagy should primarily be regarded as a stress‐adaptation and intracellular quality‐control process, whereas autophagy‐dependent cell death requires separate experimental confirmation. Natural compounds have attracted considerable attention because they may influence inflammatory signaling, oxidative stress, mitochondrial function, endothelial integrity, and PCD‐related pathways [10]. However, most evidence remains preclinical, and apparent protection in cell or animal models should not be equated with clinical efficacy. Important uncertainties remain regarding target specificity, pharmacokinetics, formulation, dose exposure, treatment timing, and reproducibility.

This review was designed around the three major clinical themes of sepsis: epidemiology, pathophysiology, and management. We first summarize recent global, regional, and Chinese epidemiological evidence and discuss the methodological limitations that affect comparisons across studies. We then examine the major pathophysiological processes of sepsis, including excessive inflammation, immunoparalysis, immunometabolic dysfunction, endothelial and coagulation abnormalities, gut dysbiosis, mitochondrial injury, and regulated cell‐death pathways. The management section focuses on guideline‐concordant antimicrobial therapy, source control, fluid and vasopressor treatment, corticosteroids, organ support, and phenotype‐guided immunomodulation, followed by a separate summary of relevant clinical trials. Recent biomarker‐guided immunotherapy, such as the ImmunoSep trial, illustrates the potential of phenotype‐based treatment while also emphasizing that improvement in a prespecified Sequential Organ Failure Assessment (SOFA)‐based endpoint does not necessarily establish a mortality benefit [11]. Finally, we evaluate preclinical studies of natural compounds that modulate PCD, classify the strength of the available evidence, and discuss the methodological, pharmacokinetic, and translational barriers that must be addressed before clinical application. A targeted narrative literature search was conducted in PubMed, Web of Science, CNKI, and Wanfang Data for studies published from January 2000 to July 2026, with priority given to recent clinical guidelines, randomized trials, high‐quality reviews, and sepsis‐specific experimental studies relevant to epidemiology, pathophysiology, management, PCD, and natural compounds.

## 2 Epidemiology

At the international level, the GBD 2021 analysis estimated 166 million incident sepsis cases and 21.4 million sepsis‐related deaths globally in 2021 [3]. Global sepsis‐related deaths decreased from 1990 to 2019 and then increased sharply during 2020–2021; however, temporal patterns differed substantially by age group, underlying cause, and region [3]. Because these estimates are model based, they should be interpreted as population‐level estimates rather than direct surveillance counts.

At the regional level, the burden of sepsis varied substantially across geographic areas. In the EPIC III study, suspected or proven infection was present in 54% of ICU patients from 88 countries, and the in‐hospital mortality rate among infected patients was 30% [12]. Infection prevalence ranged from 43% in Australasia to 60% in Asia and the Middle East [12]. These differences may reflect variations in patient characteristics, healthcare resources, ICU admission criteria, treatment practices, and case ascertainment, rather than biological differences alone.

In China, a nationwide study based on hospitalized patients reported standardized sepsis incidence rates of 328.25, 359.26, and 421.85 cases per 100,000 population in 2017, 2018, and 2019, respectively [4]. Older adults accounted for 57.5% of hospitalized cases, and the incidence was higher in northwestern China than in southeastern China [4]. The proportion of sepsis‐related deaths occurring in hospital increased from 27.0% in 2017 to 30.0% in 2019, but this measure should not be interpreted as population‐level case fatality [4]. A separate GBD 2021‐based analysis estimated 6,773,717 incident cases and 2,480,000 sepsis‐related deaths in China in 2021 [13]. GBD 2021 demonstrated substantial regional heterogeneity, with particularly high sepsis‐related mortality burdens in several sub‐Saharan African regions [3]. Although EPIC III was not a population‐based sepsis surveillance study, it provides complementary evidence of the heterogeneous burden of infection among ICUs across regions [13]. The differences between these estimates reflect the use of different populations, case definitions, data sources, and ascertainment methods.

Comparable country‐level surveillance data for 2022–2026 remain limited. Therefore, post‐2021 global incidence and mortality should not be extrapolated without standardized population‐based data. Future epidemiological studies should clearly report the study population, case definition, observation period, data source, and ascertainment method. Selected recent epidemiological evidence for sepsis is summarized in Table 1.

**TABLE 1:** Selected recent epidemiological evidence for sepsis.

Metric	Period/source	Finding	Interpretive note	References
Global estimate	GBD 2021; 1990–2021	Approximately 166 million sepsis cases and 21.4 million sepsis‐related deaths were estimated globally in 2021.	Model‐based estimates provide global context but are not equivalent to real‐time surveillance counts	[3]
International temporal trend	GBD 2021; 1990–2021	Sepsis incidence and infectious‐cause sepsis mortality generally declined until 2019, followed by an increase during 2020–2021.	COVID‐19 and changes in diagnosis and coding may affect temporal interpretation	[3]
China hospitalized burden	2017–2019; nationwide hospital data	9,455,279 patients, 10,682,625 implicit‐coded sepsis admissions, and 806,728 sepsis‐related deaths were identified; standardized incidence increased from 328.25 to 421.85 per 100,000 population.	Hospital‐based estimates are influenced by referral patterns, coding, healthcare access, and case ascertainment	[4]
China regional and age distribution	2017–2019; 31 provinces	Approximately 57.5% of hospitalized cases occurred in individuals older than 65 years; incidence was higher in northwestern China than in southeastern China.	Regional differences may reflect healthcare access and reporting practices in addition to disease burden	[4]
China in‐hospital mortality share	2017–2019	The proportion of sepsis‐related deaths occurring in hospital increased from 27.0 to 30.0%.	This measure is not equivalent to population‐level case fatality or overall sepsis mortality	[4]
Regional ICU burden	EPIC III; 2017; 88 countries	Suspected or proven infection occurred in 54% of ICU patients; in‐hospital mortality among infected patients was 30%; infection prevalence ranged from 43 to 60% across regions.	ICU infection cohort; not equivalent to mortality among all patients with sepsis	[12]
China modeled national burden	GBD 2021‐based analysis; 2021	Estimated 6,773,717 incident cases and 2,480,000 sepsis‐related deaths in China.	Reconstructed modeled estimates; not directly interchangeable with hospitalized‐sepsis data	[13]
Data gap	2022–2026	Comparable, standardized country‐level surveillance estimates remain limited after the GBD 2021 observation window.	Avoid extrapolating postpandemic trends without direct population‐based evidence	[3, 13]

## 3 Pathophysiology

Sepsis is a dynamic syndrome caused by a dysregulated host response to infection. Its pathophysiology involves interacting abnormalities in innate and adaptive immunity, endothelial function, coagulation, metabolism, and organ homeostasis. PCD is an important component of this network: excessive or mistimed cell death can remove immune cells, injure endothelial and parenchymal cells, and release DAMPs that amplify inflammation [14]. Hyperinflammation and immunosuppression may coexist in different tissues or evolve at different rates. Consistent with this view, multicohort transcriptomic analyses have identified distinct myeloid and lymphoid immune‐dysregulation endotypes that are associated with disease severity and mortality [15].

PAMPs and DAMPs are sensed by pattern‐recognition receptors and activate nuclear factor κB (NF‐κB), inflammasomes, and other inflammatory pathways. In parallel, mitochondrial injury can release mitochondrial DNA (mtDNA), which activates the cyclic GMP–AMP synthase‐stimulator of interferon genes (cGAS–STING) pathway. In experimental sepsis, STING signaling promotes gasdermin D (GSDMD)‐dependent tissue‐factor release and systemic coagulation, whereas neutrophil extracellular trap (NET)–STING signaling aggravates endothelial injury and immunothrombosis [16, 17, 18]. These mechanisms connect inflammation, oxidative stress, endothelial dysfunction, coagulation, and organ injury, and position cGAS–STING as an upstream node linking cytosolic–DNA sensing to inflammatory PCD. The dominant downstream death modality remains dependent on cell type, stimulus, and disease stage.

The following subsections first summarize excessive inflammation, oxidative stress, immunoparalysis, immunometabolic dysfunction, and gut dysbiosis. We then describe the major PCD modalities, including apoptosis, autophagy‐related cell death, pyroptosis, necroptosis, ferroptosis, cuproptosis, NETosis, and PANoptosis, and their temporal and mechanistic crosstalk in sepsis.

### 3.1 Excessive Inflammatory Response

Sepsis is characterized by a dysregulated and self‐amplifying inflammatory response [19]. PAMPs and DAMPs activate Toll‐like receptors (TLRs), NOD‐like receptors, and other pattern‐recognition receptors, inducing NF‐κB activation, inflammasome assembly, and the release of TNF‐α, IL‐1β, IL‐6, and IL‐18 [19]. This response causes vasodilation, endothelial leakage, coagulation activation, and tissue injury and can interact with inflammatory PCD to promote multiple‐organ dysfunction [14, 19]. Because broad cytokine blockade has produced inconsistent clinical benefit, current research is focusing on patient stratification and proximal regulators rather than uniform suppression of inflammation.

### 3.2 Oxidative Stress

Oxidative stress is a core driver of septic pathophysiology, arising from the excessive generation of reactive oxygen and nitrogen species that overwhelm endogenous antioxidant defenses [20]. Overproduced reactive oxygen species (ROS) fuel mitochondrial dysfunction by disrupting electron transport chain integrity, impairing oxidative phosphorylation, and triggering the opening of mitochondrial permeability transition pores, which releases proapoptotic factors and amplifies cellular injury [21]. At the systemic level, oxidative stress promotes endothelial injury, microvascular dysfunction, coagulation abnormalities, and impaired tissue oxygen utilization [20, 21, 22]. Redox‐sensitive signaling involves NF‐κB, Nrf2/HO‐1, and PI3K/Akt [20, 21, 22], whereas autophagy may limit oxidative injury by removing damaged mitochondria [23]. Targeting redox imbalance remains an active area of investigation, but the clinical benefit of broad antioxidant therapy has not been consistently established.

### 3.3 Immunoparalysis

Immunoparalysis is a common but heterogeneous state in sepsis and should not be regarded as a uniform stage that invariably follows hyperinflammation [15, 24]. This state is driven by several interrelated mechanisms, including massive apoptosis of lymphocytes and dendritic cells, sustained upregulation of negative costimulatory molecules (e.g., PD‑1, CTLA‑4), functional exhaustion of T cells, and expansion of immunosuppressive regulatory T cells and myeloid‑derived suppressor cells [25, 26]. Complementing these cellular defects, a reprogramming toward an anti‑inflammatory cytokine milieu driven by excessive IL‑10 secretion further perpetuates immune paralysis [27]. Because immune endotypes differ across patients and tissues, biomarker‐guided selection will be essential for future immunomodulatory therapies [15].

### 3.4 Immunometabolic Dysfunction

Parallel to the inflammatory and oxidative alterations, sepsis is characterized by profound immunometabolic dysfunction that reshapes immune cell function and systemic homeostasis. Leukocytes from septic patients and animal models exhibit a distinct metabolic shift, including enhanced aerobic glycolysis, impaired mitochondrial oxidative phosphorylation, and altered lipid metabolism. This metabolic rewiring not only underpins the functional polarization of macrophages toward a hyperinflammatory M1 phenotype but also contributes to immune cell exhaustion and organ damage. For example, forced reliance on glycolysis in proinflammatory macrophages fuels NLRP3 inflammasome activation and cytokine production, while defective fatty acid oxidation compromises the survival and reparative capacity of anti‑inflammatory M2 macrophages. The emerging field of immunometabolism thus offers a new conceptual framework for understanding sepsis pathophysiology and a rich source of potential biomarkers and therapeutic targets [28, 29, 30].

### 3.5 Gut Dysbiosis

Accumulating evidence indicates that gut dysbiosis contributes to sepsis pathophysiology rather than acting as a passive consequence of critical illness. Critical illness and sepsis are associated with disruption of intestinal and respiratory microbial communities, including enrichment of the lung microbiome with gut‐associated bacteria [31, 32, 33]. Sepsis‐associated dysbiosis is characterized by reduced microbial diversity, expansion of pathobionts, and depletion of beneficial commensals [32, 33]. These changes may impair intestinal epithelial integrity and facilitate the systemic dissemination of bacteria, lipopolysaccharide (LPS), and other microbial products. Dysbiosis‐related reductions in short‐chain fatty acid production and alterations in mucosal immunity may further compromise host defense and delay recovery [34, 35]. Biomarkers of gut injury and microbiome‐based interventions, including fecal microbiota transplantation, targeted probiotics, and metabolic modulation, remain investigational and require standardized clinical validation [36].

### 3.6 Programmed Cell Death (PCD)

PCD comprises a heterogeneous set of biological processes that support tissue homeostasis through the elimination of damaged or harmful cells. Major PCD modalities include traditional forms (apoptosis and autophagy), inflammatory forms (pyroptosis and necroptosis), metabolic forms (ferroptosis and cuproptosis), a specialized form (NETosis), and an integrated form (PANoptosis). In sepsis, dysregulated apoptosis contributes to immunosuppression; pyroptosis drives cytokine storms; ferroptosis induces metabolic collapse; and impaired autophagic flux exacerbates organ injury. PCD pathways are interconnected rather than isolated. Although some studies suggest that lytic inflammatory cell death may predominate during early injury and apoptotic or metabolic cell death may become more evident later, these patterns are context dependent and should not be interpreted as a fixed temporal sequence [14, 15]. Dysregulated PCD can remove immune cells, damage endothelial and parenchymal cells, release DAMPs, and amplify systemic inflammation. In summary, the detailed pathophysiological mechanisms of sepsis are illustrated in Figure 1.

**FIGURE 1:**
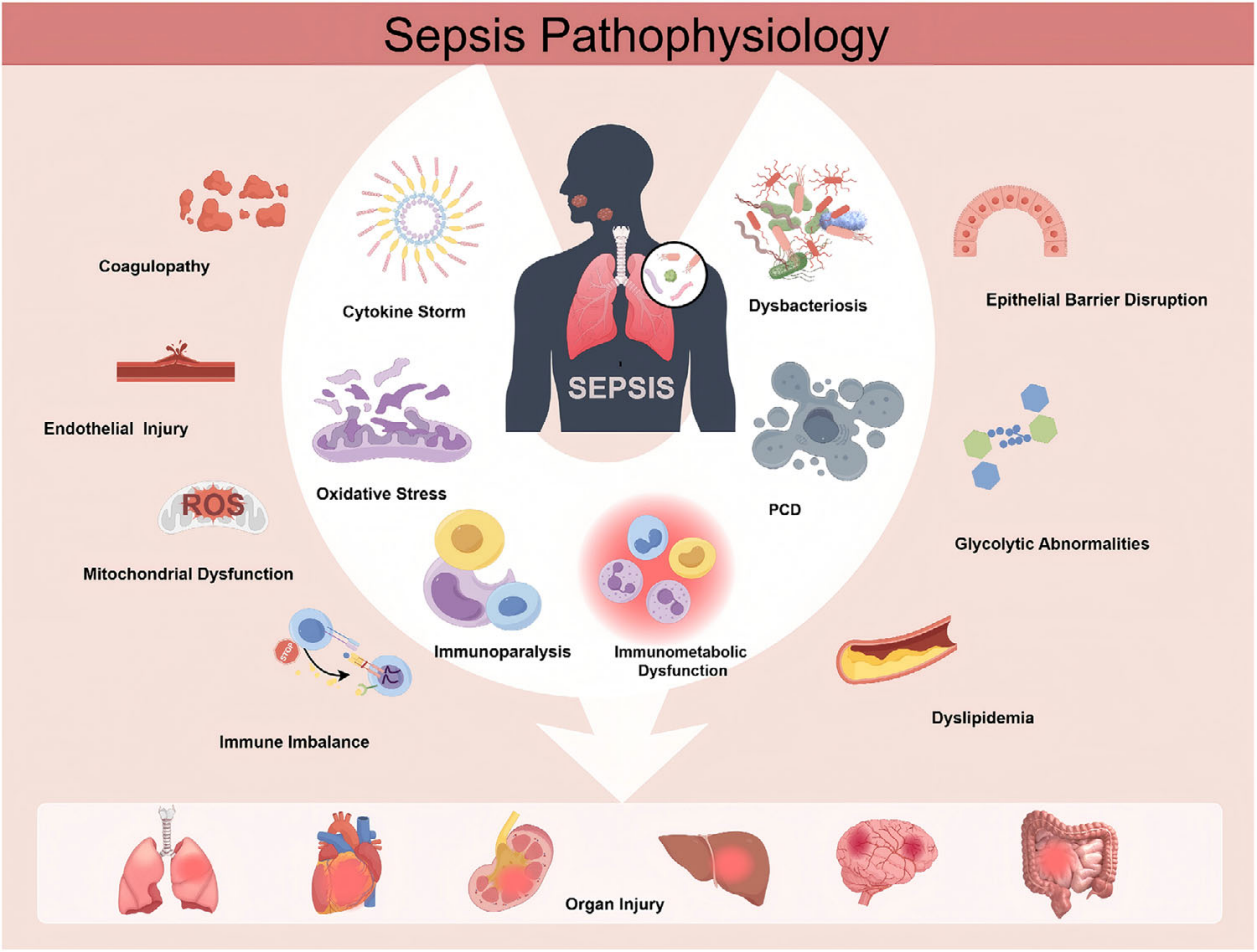
Pathophysiological mechanisms of sepsis. ① The core pathophysiological mechanisms primarily including excessive inflammatory response (cytokine storm), oxidative stress, immunoparalysis, immunometabolic dysfunction, dysbacteriosis, and PCD. ② The secondary pathophysiological mechanisms primarily including coagulopathy, endothelial injury, mitochondrial dysfunction, immune imbalance, dyslipidemia, glycolytic abnormalities, and epithelial barrier disruption. ③ Sepsis ultimately leads to organ damage or MODS and even death.

#### 3.6.1 Apoptosis

Apoptosis is a regulated form of PCD characterized by intact plasma membrane structure, chromatin condensation, DNA fragmentation, cellular shrinkage, and apoptotic body formation, and it generally occurs without provoking inflammation. This process contributes to tissue homeostasis by enabling the controlled removal of damaged cells [37]. Mechanistically, apoptosis is mediated by two principal pathways: the intrinsic (mitochondrial) pathway and the extrinsic (death receptor‐dependent) pathway. The intrinsic pathway is regulated by Bcl‐2 family proteins, which control mitochondrial membrane permeability and promote cytochrome *c* release. Cytochrome *c* subsequently associates with Apaf‐1 and Procaspase‐9 to form the apoptosome, resulting in activation of effector Caspase‐3 [38, 39]. The extrinsic pathway is initiated by ligand binding to death receptors such as Fas or TNFR, leading to FADD recruitment and Caspase‐8 activation, which subsequently induces Caspase‐3 activation [40]. In addition, multiple signaling pathways, including PI3K/Akt, MAPK, p53, NF‐κB, and oxidative stress‐related pathways, modulate apoptotic sensitivity and regulate the balance between cell survival and cell death [41]. Persistent lymphocyte apoptosis is a hallmark of sepsis‐induced immunosuppression and is strongly associated with poor prognosis and secondary infections [26].

#### 3.6.2 Autophagy

Autophagy is a lysosome‐mediated intracellular degradation pathway that supports cellular homeostasis through the removal of damaged organelles, aggregated proteins, and metabolic byproducts [42]. This process involves three core stages: autophagosome formation, fusion with lysosomes, and subsequent cargo breakdown. Autophagy is initiated by activation of the ULK1 complex, followed by membrane nucleation driven by the Beclin‐1–Vps34 complex, and completed through membrane expansion and cargo incorporation mediated by LC3‐II [43]. Under physiological conditions, autophagy plays predominantly cytoprotective roles; however, excessive activation or dysregulated control can lead to autophagy‐dependent cell death, which operates independently of apoptotic pathways [44]. Moreover, autophagy modulates other forms of PCD by removing damaged mitochondria, reducing ROS, and suppressing inflammasome activation [45].

#### 3.6.3 Pyroptosis

Pyroptosis is an inflammatory form of PCD driven by caspase activation and characterized by membrane pore formation, cellular swelling, membrane disruption, and the release of IL‐1β and IL‐18, leading to strong inflammatory responses [46]. In the canonical pathway, pathogen or damage‐derived cues activate inflammasome sensors such as NLRP3 or AIM2, resulting in ASC recruitment and Caspase‐1 activation. Caspase‐1 then cleaves GSDMD, and the resulting N‐terminal fragment inserts into the plasma membrane to induce pore formation, cell rupture, and cytokine release [47, 48]. By contrast, the noncanonical pathway involves direct sensing of intracellular LPS by human Caspase‐4/5 or murine Caspase‐11, which cleave GSDMD and initiate pyroptotic cell death [49, 50]. Pyroptosis also interacts with other PCD modalities, including apoptosis and ferroptosis, and its activation is modulated by signaling inputs such as NF‐κB activation, ROS, and potassium efflux [51].

#### 3.6.4 Necroptosis

Necroptosis is a regulated form of necrotic cell death that depends on the receptor‐interacting protein kinase (RIPK) signaling pathway. Morphologically, it resembles necrosis—exhibiting cell swelling, membrane rupture, and release of intracellular contents—but is driven by defined molecular mechanisms [52]. In the canonical pathway, binding of TNF‐α to TNFR1 induces assembly of Complex I. When Caspase‐8 activity is inhibited, RIPK1 and RIPK3 undergo mutual phosphorylation and assemble into the “necrosome,” which subsequently phosphorylates mixed lineage kinase domain‐like protein (MLKL). Phosphorylated MLKL oligomerizes and translocates to the plasma membrane, where it forms ion channel‐like pores that trigger cell lysis and inflammatory responses [53, 54]. Necroptosis is further modulated by ROS, calcium flux, mitochondrial function, and immune signals such as interferons and TLRs. It also exhibits extensive crosstalk with apoptosis and pyroptosis and is critical in development, inflammation, and a wide range of disease conditions [55, 56].

#### 3.6.5 Ferroptosis

Ferroptosis is a nonapoptotic form of PCD distinguished by iron‐driven lipid peroxidation that ultimately compromises plasma membrane integrity [57]. This process is primarily regulated by the System Xc^−^–GSH–GPX4 axis. Disruption of cystine uptake via System Xc^−^ or depletion of glutathione (GSH) diminishes GPX4 activity, leading to insufficient detoxification of lipid peroxides and subsequent iron‐dependent membrane injury [58]. In parallel, accumulation of intracellular free iron enhances ROS production through Fenton chemistry, thereby exacerbating lipid peroxidation. Enzymes involved in lipid metabolism, including ACSL4 and lipoxygenases (LOX), further promote the oxidation of polyunsaturated fatty acids within cellular membranes [59]. Ferroptotic susceptibility is additionally influenced by signaling pathways such as NRF2, p53, and MAPK, and ferroptosis has been implicated in a range of pathological conditions, including inflammation, cancer, and ischemia–reperfusion injury [60, 61]. Recent studies have further elucidated the role of ferroptosis in sepsis‑induced multiorgan dysfunction, highlighting organ‑specific signatures and potential therapeutic windows [62, 63]. Ferroptosis also intersects with pyroptosis and necroptosis through shared signals involving ROS, lipid peroxidation, iron handling, and mitochondrial injury. These overlaps support investigation of upstream redox interventions but do not establish direct conversion between individual death modalities [14, 60, 61, 62, 63].

#### 3.6.6 Cuproptosis

Cuproptosis is a recently identified, copper‐dependent form of regulated cell death that is tightly linked to mitochondrial metabolism. Its core mechanism involves the direct binding of excessive copper ions to lipoylated mitochondrial proteins within the tricarboxylic acid cycle, such as dihydrolipoamide S‐acetyltransferase, resulting in their aberrant aggregation. Concurrently, copper overload promotes the loss of iron–sulfur cluster proteins, leading to proteotoxic stress and collapse of cellular energy metabolism, ultimately causing cell death [64, 65, 66]. This process requires Ferredoxin 1, which regulates protein lipoylation levels and is mechanistically distinct from classical PCD pathways, including apoptosis, ferroptosis, and necroptosis [67, 68].

#### 3.6.7 NETosis

NETosis is an inflammatory form of PCD specific to neutrophils, marked by large‐scale chromatin relaxation and the extracellular release of DNA together with antimicrobial granule proteins such as neutrophil elastase and myeloperoxidase (MPO). These components assemble into NETs, which restrict pathogen dissemination but can also exacerbate inflammation and immune‐related tissue damage [69]. Mechanistically, NET formation can occur through lytic and nonlytic routes; the term NETosis should be reserved for NET release accompanied by neutrophil death. PAD4‐mediated histone citrullination contributes to NET formation in a stimulus‐dependent manner [69, 70, 71, 72]. Beyond pathogen entrapment, excessive NETosis directly damages the endothelium, activates the coagulation cascade via platelet and thrombin generation, and promotes microvascular thrombosis, thereby exacerbating sepsis‑induced multiorgan dysfunction [73, 74]. Furthermore, NETs can also promote NLRP3 deubiquitination and inflammasome assembly in alveolar macrophages, leading to Caspase‐1‐dependent pyroptosis and aggravating experimental septic lung injury [72].

#### 3.6.8 PANoptosis

PANoptosis is an integrated mode of PCD induced by inflammatory cues, infection, or cellular stress and is defined by the simultaneous activation of pyroptosis, apoptosis, and necroptosis, which function in a coordinated and compensatory manner through a multiprotein complex termed the PANoptosome [75, 76]. Under specific stimuli, such as combined TNF‐α and IFN‐γ exposure, viral infection, or pathogen invasion, key molecules, including RIPK1, RIPK3, ASC, Caspase‐8, and Caspase‐1, interact to assemble the PANoptosome, thereby engaging multiple death pathways and promoting the release of proinflammatory cytokines that drive cell lysis and amplify inflammation [77, 78]. The principal biological significance of PANoptosis lies in its signaling redundancy and immune‐protective properties. When one death pathway is inhibited, alternative pathways can be compensatorily activated, ensuring an effective host defense against infection and cellular stress [79]. Upstream signals triggering PANoptosome assembly include ZBP1 sensing of viral or bacterial nucleic acids, AIM2 recognition of cytosolic DNA, and inflammatory cytokines such as TNF‑α and IFN‑γ [77, 80]. Emerging evidence indicates that PANoptosis is actively engaged in sepsis‑induced acute lung injury (ALI) and myocardial dysfunction, with novel regulators such as DLL4^+^ neutrophils and zinc homeostasis being identified [80, 81]. In summary, the core mechanisms of major PCD pathways in sepsis are presented in Figure 2.

**FIGURE 2:**
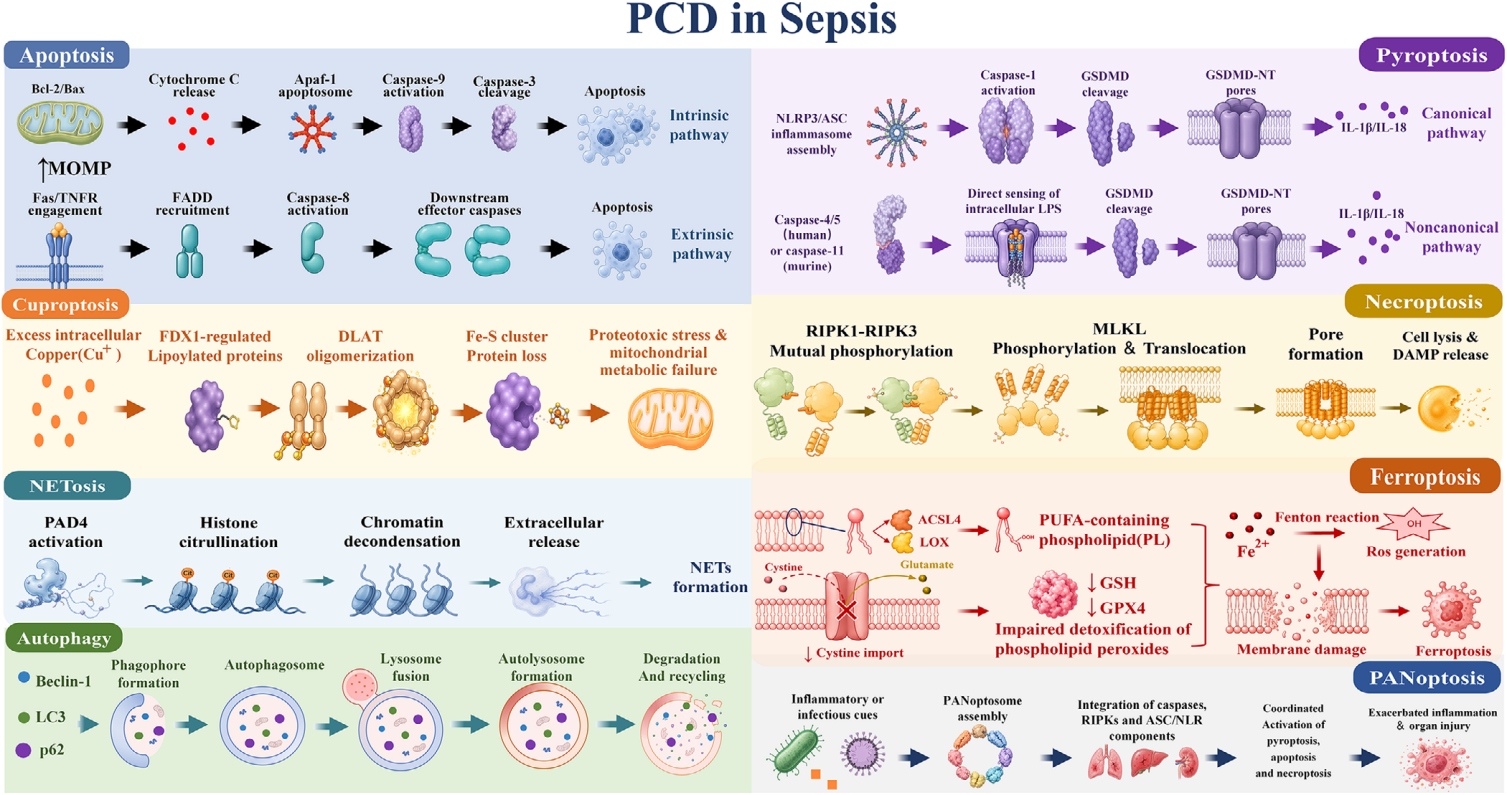
Core mechanisms of major PCD pathways in sepsis: apoptosis: ① apoptosis: intrinsic pathway: Bcl‐2/Bax‐mediated increase in mitochondrial outer membrane permeability → cytochrome *c* release → Apaf‐1 apoptosome formation → Caspase‐9 activation → Caspase‐3 cleavage; extrinsic pathway: Fas/TNFR engagement → FADD recruitment → Caspase‐8 activation → downstream effector caspases; ② autophagy: autophagy initiation and progression involving Beclin‐1, LC3 conversion (LC3‐I → LC3‐II), and p62‐mediated cargo recognition, followed by autophagosome–lysosome fusion and degradation; ③ pyroptosis: canonical pathway: NLRP3/ASC inflammasome assembly → Caspase‐1 activation → cleavage of pro‐IL‐1β/pro‐IL‐18 and GSDMD → formation of GSDMD‐NT membrane pores; noncanonical pathway: human Caspase‐4/5 or murine Caspase‐11 directly sense intracellular LPS and cleave GSDMD; ④ necroptosis: RIPK1–RIPK3 mutual phosphorylation → MLKL phosphorylation and translocation to the plasma membrane → pore formation → cell lysis and DAMP release; ⑤ ferroptosis: iron‐dependent lipid peroxidation driven by dysfunction of the System Xc^−^(SLC7A11)–GSH–GPX4 axis; ACSL4 and LOX promote PUFA peroxidation, while Fe^2^^+^ catalyzes ROS generation via the Fenton reaction; ⑥ cuproptosis: excess intracellular copper binds FDX1‐regulated lipoylated proteins → induces DLAT oligomerization and Fe–S cluster protein loss → proteotoxic stress → mitochondrial metabolic failure; ⑦ NETosis: PAD4‐mediated histone citrullination → chromatin decondensation and extracellular release → formation of NETs (DNA + histones + granular proteins) for pathogen trapping; ⑧ PANoptosis: under specific inflammatory or infectious cues, pyroptosis, apoptosis, and necroptosis are coordinately activated via the PANoptosome, integrating caspases, RIPKs, and ASC/NLR components.

In summary, sepsis should be regarded as a highly dynamic and interconnected syndrome rather than a simple infection‐driven inflammatory disorder. Excessive inflammation, oxidative stress, immunoparalysis, immunometabolic dysfunction, gut dysbiosis, and multiple PCD pathways collectively form an integrated pathogenic network mediated by shared signaling hubs such as NF‐κB, ROS, mitochondrial dysfunction, and inflammasome activation. Importantly, different PCD modalities exhibit substantial temporal and mechanistic crosstalk throughout sepsis progression, jointly shaping immune dysregulation, tissue injury, and multiorgan failure. These advances have shifted the current understanding of sepsis from isolated inflammatory events toward a systems‐level collapse of immune‐metabolic homeostasis. Consequently, therapeutic strategies targeting coordinated pathogenic networks and dysregulated PCD pathways may provide more effective approaches for improving outcomes in sepsis. The following sections therefore summarize current and emerging therapeutic interventions targeting these interconnected mechanisms.

## 4 Management

The clinical management of sepsis has evolved considerably over the past two decades, transitioning from largely supportive care toward a structured, evidence‐based framework centered on early recognition, rapid antimicrobial administration, hemodynamic stabilization, and timely source control [82, 83, 84]. Despite these advances, sepsis remains a major global cause of morbidity and mortality [85], while effective host‐directed therapies remain limited [86].

### 4.1 Antimicrobial Therapy and Source Control

Prompt administration of appropriate antimicrobials remains a cornerstone of sepsis treatment. The 2026 Surviving Sepsis Campaign recommends immediate antimicrobial therapy, ideally within 1 h of recognition, for patients with septic shock and for those with probable or definite sepsis without shock. For patients with possible sepsis without shock, a time‐limited rapid investigation is appropriate, with antimicrobial therapy administered within 3 h if concern for infection persists [5]. Blood cultures should be obtained as early as possible and preferably before antimicrobial administration, provided that this does not delay treatment [5]. At the level of β‐lactam administration, the BLING III trial found no statistically significant reduction in 90‐day mortality with continuous compared with intermittent infusion [87]. Patients should be rapidly evaluated for anatomical sources requiring intervention. When source control is indicated, drainage, debridement, removal of infected devices, or other definitive procedures should be undertaken early, ideally within 6 h of diagnosis whenever clinically feasible [5, 88, 89].

### 4.2 Hemodynamic Management

Hemodynamic support in sepsis integrates timely fluid resuscitation with early vasopressor therapy to restore tissue perfusion and maintain hemodynamic stability. Current guidelines suggest administering at least 30 mL/kg of intravenous crystalloid within the first 3 h for sepsis‐induced hypoperfusion or septic shock, while emphasizing frequent reassessment and individualization according to patient characteristics, perfusion status, and the risk of fluid overload [5]. After initial resuscitation, the CLOVERS and CLASSIC trials found no significant mortality difference between restrictive and comparator fluid strategies, underscoring the importance of individualized fluid administration and ongoing reassessment [90, 91]. Dynamic measures, including passive leg raising, may help assess fluid responsiveness [92]. Balanced crystalloids are preferred over 0.9% saline for initial resuscitation [5, 93]. Norepinephrine remains the first‐line vasopressor, with an initial mean arterial pressure target of approximately 65 mmHg [5]. In patients with septic shock, vasopressors may be initiated through peripheral intravenous access when this avoids delaying treatment, with contemporary evidence indicating a low incidence of major adverse events during appropriately monitored short‐term peripheral administration [5, 94]. Early norepinephrine administration may improve early shock control, although a significant mortality benefit has not been established [95]. When norepinephrine requirements escalate, vasopressin may be added, followed by epinephrine if an adequate mean arterial pressure cannot be maintained [5]. For patients with septic shock, intravenous corticosteroids may be considered as adjunctive therapy. ADRENAL demonstrated faster shock resolution without a significant reduction in 90‐day mortality, whereas APROCCHSS reported lower 90‐day mortality with hydrocortisone plus fludrocortisone [96, 97].

### 4.3 Organ Support and Supportive Care

Organ support remains a central component of sepsis management and should be individualized according to the type and severity of organ dysfunction. In patients with sepsis‐associated acute hypoxemic respiratory failure, high‐flow nasal oxygen may be used when appropriate. For patients with sepsis‐associated acute respiratory distress syndrome (ARDS), lung‐protective ventilation using a low tidal volume of approximately 6 mL/kg predicted body weight and limitation of plateau pressure to ≤30 cm H_2_O is recommended. Prone positioning for more than 12 h per day should be considered in patients with moderate‐to‐severe ARDS, whereas venovenous extracorporeal membrane oxygenation may be considered for severe refractory respiratory failure in experienced centers [5]. Renal replacement therapy should not be initiated solely on the basis of acute kidney injury in the absence of established indications; when renal replacement therapy is required, either continuous or intermittent modalities may be selected according to the patient's hemodynamic status and clinical context [5].

Supportive care should be integrated with organ‐specific treatment and includes a restrictive red‐blood‐cell transfusion strategy, early enteral nutrition when feasible, glycemic control, and prevention of venous thromboembolism. Enteral nutrition should generally be initiated within 72 h when oral intake is not feasible and no contraindication exists. Insulin therapy is recommended when blood glucose levels are ≥180 mg/dL, while pharmacological venous thromboembolism prophylaxis, preferably with low‐molecular‐weight heparin when not contraindicated, should be routinely considered [5]. These supportive measures should be accompanied by regular reassessment of fluid balance, nutritional status, mobility, and rehabilitation needs throughout the course of critical illness.

### 4.4 Immune‐Directed Therapies

Immune‐directed therapy in sepsis remains investigational and should not currently be considered routine treatment. Immune‐stimulatory approaches such as GM‐CSF, IL‐7, and interferon‐γ, as well as immune‐checkpoint modulation, have been explored for sepsis‐associated immunoparalysis, but their efficacy appears highly dependent on immune phenotype, disease stage, and patient selection [98, 99].

The recent ImmunoSep trial provides proof of concept for a phenotype‐guided strategy. Patients with macrophage activation‐like syndrome received anakinra, whereas those with sepsis‐induced immunoparalysis received recombinant human interferon‐γ. Precision immunotherapy improved the prespecified SOFA‐based organ‐dysfunction endpoint by Day 9, but did not significantly reduce 28‐day mortality [11]. These findings support further development of biomarker‐guided immune modulation rather than indiscriminate immune stimulation or suppression.

### 4.5 Adjunctive and Advanced Therapies

Adjunctive and advanced therapies have been extensively investigated in sepsis, but most have not demonstrated sufficiently consistent benefit for routine clinical use. The 2026 Surviving Sepsis Campaign suggests against routine intravenous vitamin C, intravenous immunoglobulin, and extracorporeal blood‐purification techniques, including hemoperfusion, high‐dose hemofiltration, plasma exchange, and polymyxin B hemoperfusion [5, 100, 101]. These recommendations illustrate the persistent gap between strong biological rationale and reproducible patient‐centered benefit. Other host‐directed approaches, including cellular therapies and novel innate‐immune modulators, remain investigational and should primarily be evaluated within well‐designed clinical trials.

### 4.6 Precision Medicine

Although protocolized care bundles have improved early recognition and standardized management, the biological heterogeneity of sepsis limits a uniform “one‐size‐fits‐all” approach [82, 84, 102, 103]. Increasing evidence indicates that sepsis comprises dynamically evolving clinical, immune, endothelial, metabolic, and transcriptomic subphenotypes or endotypes that may differ in prognosis and treatment response [15, 84, 103, 104]. Accordingly, contemporary sepsis research is shifting toward precision medicine approaches that integrate clinical phenotyping, biomarker profiling, transcriptomics, and immune phenotyping to guide therapeutic enrichment and individualized interventions [24, 99, 102, 104]. However, no universally validated endotype‐guided treatment algorithm is currently established for routine sepsis care, and prospective validation remains essential [84, 102, 103, 104]. The principal contemporary management strategies for sepsis are summarized in Table 2, whereas representative randomized clinical trials informing these approaches are discussed separately in Section 4.7 and Table 3.

**TABLE 2:** Summary of contemporary management strategies for sepsis.

Management domain	Treatment strategy	Key clinical considerations	References
Antimicrobial therapy	Prompt administration of appropriate antimicrobial therapy	Initiate antimicrobial therapy as early as possible according to the likelihood and severity of infection. Blood cultures should preferably be obtained before antimicrobial administration when this does not delay treatment. Antimicrobial therapy should subsequently be adjusted according to microbiological findings and clinical response.	[5]
Source control	Early identification and elimination of the infectious source	Drainage of infected collections, debridement, removal of infected devices, or other definitive source‐control procedures should be performed as early as clinically feasible.	[5, 88, 89]
Fluid resuscitation	Initial crystalloid resuscitation followed by individualized reassessment	Intravenous crystalloids are recommended for sepsis‐induced hypoperfusion or septic shock. Subsequent fluid administration should be individualized according to perfusion status, fluid responsiveness, and the risk of fluid overload. Balanced crystalloids are preferred over 0.9% saline for initial resuscitation.	[5, 92, 93]
Vasopressor therapy	Early vasopressor support to restore adequate perfusion pressure	Norepinephrine is the preferred first‐line vasopressor. An initial mean arterial pressure target of approximately 65 mmHg is generally appropriate. Vasopressin may be added when norepinephrine requirements increase, followed by epinephrine if adequate blood pressure cannot be maintained.	[5, 94, 95]
Corticosteroids	Adjunctive corticosteroid therapy in selected patients with septic shock	Intravenous corticosteroids may be considered in patients with septic shock who have persistent vasopressor requirements despite adequate resuscitation.	[5]
Respiratory support	Lung‐protective respiratory support for sepsis‐associated respiratory failure	High‐flow nasal oxygen may be used in appropriate patients with acute hypoxemic respiratory failure. For sepsis‐associated ARDS, lung‐protective ventilation with a tidal volume of approximately 6 mL/kg predicted body weight and plateau pressure ≤30 cmH_2_O is recommended. Prone positioning should be considered in moderate‐to‐severe ARDS, while venovenous extracorporeal membrane oxygenation may be considered for refractory respiratory failure in experienced centers.	[5]
Renal support	Renal replacement therapy when established indications are present	Renal replacement therapy should not be initiated solely on the basis of acute kidney injury. When required, continuous or intermittent modalities may be selected according to hemodynamic status and the overall clinical context.	[5]
Transfusion and metabolic support	Restrictive transfusion strategy and controlled metabolic support	Supportive care includes a restrictive red‐blood‐cell transfusion strategy, glycemic control, and repeated reassessment of metabolic and hemodynamic status. Insulin therapy is recommended when blood glucose concentrations reach the treatment threshold defined by current guidelines.	[5]
Nutritional support	Early enteral nutrition when clinically feasible	Enteral nutrition should generally be initiated early when oral intake is not feasible and no contraindication exists, while nutritional status should be reassessed throughout critical illness.	[5]
Venous thromboembolism prophylaxis	Pharmacological thromboprophylaxis	Pharmacological prophylaxis should be routinely considered in the absence of contraindications, with low‐molecular‐weight heparin generally preferred.	[5]
Immune‐directed therapy	Phenotype‐guided immune modulation in selected patients	Immune‐stimulatory approaches, including GM‐CSF, IL‐7 and interferon‐γ, as well as immune‐checkpoint modulation, remain investigational and should not be used routinely outside carefully selected patients or clinical studies.	[98, 99]
Adjunctive and advanced therapies	Restrict nonestablished adjunctive therapies to selected settings	Routine intravenous vitamin C, intravenous immunoglobulin, and extracorporeal blood‐purification strategies are not recommended for routine use. Cellular therapies and novel innate‐immune modulators remain investigational.	[5, 100, 101]
Precision management	Individualized treatment based on clinical phenotype and biological endotype	Clinical, immune, endothelial, metabolic, and transcriptomic phenotyping may help identify biologically distinct patient subgroups and support therapeutic enrichment. However, no universally validated endotype‐guided treatment algorithm is currently available for routine clinical practice.	[15, 24, 82, 84, 99, 102, 103, 104]

**TABLE 3:** Selected randomized clinical trials informing contemporary sepsis management.

Domain	Trial/NCT No.	Phase/design and status	Intervention	Main objective	Key finding	References
Precision immunotherapy	ImmunoSep, NCT04990232	Phase 2b RCT; completed	Anakinra or rhIFN‐γ selected by immune phenotype	Test biomarker‐guided immunotherapy	Improved Day‐9 SOFA‐based endpoint; no significant 28‐day mortality benefit	[11]
Antimicrobial therapy	BLING III, NCT03213990	Phase III RCT; completed	Continuous vs. intermittent β‐lactam infusion	Determine whether continuous infusion reduces 90‐day mortality	90‐day mortality 24.9 vs. 26.8%; primary difference did not reach statistical significance	[87]
Hemodynamic management	CLOVERS, NCT03434028	Phase III RCT; completed	Restrictive fluids/earlier vasopressors vs. liberal fluids	Compare early fluid/vasopressor strategies in sepsis‐induced hypotension	No significant difference in death before discharge home by Day 90	[90]
Hemodynamic management	CLASSIC, NCT03668236	Multicenter strategy RCT; completed	Restrictive vs. standard IV‐fluid therapy	Assess benefits and harms of fluid restriction in ICU septic shock	90‐day mortality 42.3 vs. 42.1%; no significant difference	[91]
Corticosteroids	ADRENAL, NCT01448109	Multicenter RCT; completed	Hydrocortisone 200 mg/day vs. placebo	Assess 90‐day mortality in septic shock	No significant 90‐day mortality reduction; faster shock reversal	[96]
Corticosteroids	APROCCHSS, NCT00625209	Multicenter RCT; completed	Hydrocortisone + fludrocortisone vs. placebo	Assess mortality in septic shock	Lower 90‐day mortality: 43.0 vs. 49.1%	[97]

### 4.7 Selected Clinical Trials

Several landmark randomized trials have refined contemporary sepsis management by evaluating antimicrobial administration, fluid and vasopressor strategies, corticosteroids, and phenotype‐guided immunotherapy. Importantly, these studies have more often refined how established therapies should be delivered than identified universally effective new treatments. Selected trials and their clinical implications are summarized in Table 3.

Collectively, these trials underscore that treatment effects in sepsis are frequently modest or phenotype dependent, reinforcing the importance of individualized treatment while avoiding premature extrapolation from mechanistic or early‐phase evidence.

## 5 Experimental Natural Compounds Targeting PCD

The following section focuses specifically on experimental natural compounds that modulate PCD‐associated pathways in preclinical sepsis models. Unless otherwise stated, the evidence summarized here is derived predominantly from cellular or animal studies and should not be interpreted as demonstrating clinical efficacy in patients with sepsis. Pathway‐specific evidence, experimental models, molecular targets, and major methodological limitations are summarized in the Table 4.

**TABLE 4:** Natural compounds targeting PCD In sepsis: mechanisms and signaling pathways.

PCD	Natural compound	Molecular formula	2D structure	Mechanisms of action	Targets/pathways	Evidence level	References
Apoptosis	Resveratrol	C_14_H_12_O_3_	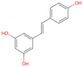	Inhibits NF‐κB activation, enhances Nrf2 signaling, reduces proinflammatory cytokines (e.g., TNF‐α) and oxidative stress, improves microcirculation, and attenuates multiple organ injury in sepsis	Sirt1/AMPK/Nrf2	In vivo	[106]
	Curcumin	C_21_H_20_O_6_	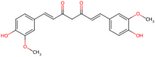	Suppresses NF‐κB, PI3K/Akt, and MAPK signaling; reduces cytokine release and oxidative stress; regulates Bcl‐2/Bax balance; inhibits apoptosis and ameliorates sepsis pathology	NF‐κB/ MAPK	In vivo; clinical randomized controlled trial	[107, 108]
	Paeoniflorin	C_23_H_28_O_11_	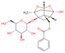	Activates Nrf2/Keap1 axis, enhances antioxidant defense, suppresses inflammation and apoptosis, and alleviates sepsis‐induced acute lung injury	Nrf2/HO‐1	In vivo	[109]
	Quercetin	C_15_H_10_O_7_	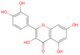	Inhibits NF‐κB, activates Nrf2, modulates Bcl‐2/Bax, reduces inflammation and oxidative stress, and limits apoptosis during sepsis	PI3K/Akt	In vivo + in vitro	[110]
	Chlorogenic acid	C_16_H_18_O_9_	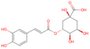	Suppresses NF‐κB and NLRP3 inflammasome activation; reduces inflammation; regulates Bcl‐2/Bax balance; inhibits apoptosis	Nrf2/HO‐1	In vivo	[111]
	Ginsenosides	C_42_H_72_O_13_	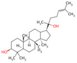	Modulate PI3K/Akt and NF‐κB pathways; suppress inflammation; regulate Bcl‐2/Bax expression; inhibit apoptosis and protect against organ dysfunction	PI3K/Akt	In vivo	[112]
	Tanshinone IIA	C_19_H_18_O_3_	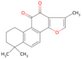	Inhibits NF‐κB, activates Nrf2, decreases oxidative stress and inflammation, regulates Bcl‐2/Bax, and suppresses apoptosis	NF‐κB/MAPK	In vivo	[113]
	Allicin	C_6_H_10_OS_2_	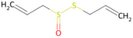	Activates the Nrf2/HO‐1 pathway, inhibits NLRP3 inflammasome activity, reduces inflammation, and suppresses apoptosis in sepsis models	Nrf2/HO‐1, NLRP3	In vitro	[114]
	Celastrol	C_29_H_38_O_4_	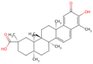	Inhibits MAPK and PI3K/Akt signaling, reduces inflammation, apoptosis, and oxidative stress, and protects against LPS‐induced acute liver injury	MAPK, PI3K/AKT	In vivo	[115]
	Byakangelicin	C_17_H_18_O_7_	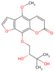	Reduces sepsis‐induced acute kidney injury by inhibiting inflammation and apoptosis; may act via suppression of NF‐κB and regulation of the proteasome subunit PSMC5	NF‐κB, PSMC5	In vivo	[116]
Pyroptosis	Chitosan oligosaccharides	C_12_H_24_N_2_O_9_	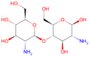	Activates the Nrf2/GPX4 antioxidant pathway, suppresses ROS production, inhibits Caspase‐1/GSDMD cleavage, reduces macrophage pyroptosis, and improves sepsis outcomes	Nrf2/GPX4, Caspase‐1/GSDMD	In vivo	[121]
	Corilagin	C_27_H_22_O_18_	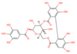	Binds to the TIR domain of MyD88 and the CARD domain of ASC, disrupts NLRP3 inflammasome assembly, and suppresses GSDMD cleavage	MyD88/NF‐κB/NLRP3/ASC/GSDMD	In vitro + in vivo	[121]
	Americanin B	C_27_H_24_O_9_	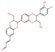	Inhibits NLRP3 inflammasome activation and downregulates Caspase‐1 and GSDMD expression	NLRP3/Caspase‐1/GSDMD	In vitro	[122]
	Sinapic Acid	C_11_H_12_O_5_	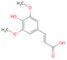	Selectively suppresses NLRP3 inflammasome activation, blocks Caspase‐1 activation and IL‐1β secretion, and inhibits ASC oligomerization	NLRP3/Caspase‐1/IL‐1β/ASC	In vivo	[123]
	Dihydromyricetin	C_15_H_12_O_8_	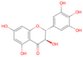	Inhibits NLRP3/ASC/Caspase‐1 signaling, reduces GSDMD expression and cleavage, decreases IL‐1β/IL‐18 release, and ameliorates sepsis‐induced acute lung injury	NLRP3/ASC/Caspase‐1	In vitro	[124]
	Phillyrin	C_27_H_34_O_11_	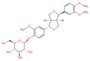	Suppresses NLRP3–Caspase‐1–GSDMD‐dependent pyroptosis, reduces ASC speck formation and GSDMD‐N generation, and protects against sepsis‐associated acute lung injury	NLRP3–Caspase‐1–GSDMD	In vitro	[125]
Ferroptosis	Andrographolide	C_20_H_30_O_5_	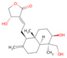	Enhances antioxidant defenses via activation of the Nrf2/FSP1 axis; regulates the Keap1/Nrf2 pathway to reduce inflammation and ferroptosis, protecting renal and pulmonary tissues	Nrf2/FSP1, Keap1/Nrf2	In vitro	[129, 131]
	Ginsenoside Rg1	C_42_H_72_O_14_	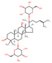	Inhibits tubular epithelial ferroptosis and reduces lipid peroxidation; activates antioxidant defenses via the m^6^A‐dependent PGC‐1α/Nrf2 pathway	Nrf2, PGC‐1α/Nrf2	In vivo	[130, 132]
	Hyperoside	C_21_H_20_O_12_	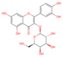	Activates Nrf2 signaling, upregulates antioxidant proteins such as GPX4, directly suppresses ferroptosis, and mitigates oxidative lung injury	Nrf2	In vivo	[133]
	Baicalein	C_15_H_10_O_5_	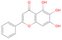	Regulates the miR‐299b‐5p/HIF‐1α axis to inhibit ferroptosis‐related oxidative damage and protect cardiomyocytes in sepsis	miR‐299b‐5p/HIF1‐α	In vivo	[134]
	Chrysin	C_15_H_10_O_4_	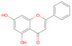	Upregulates Nrf2‐dependent antioxidant responses (e.g., GPX4, HO‐1), suppressing LPS‐induced ferroptosis and oxidative stress in lung tissue	Nrf2	In vivo	[135]
	Isoferulic acid	C_10_H_10_O_4_	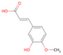	Activates SIRT1 signaling to inhibit hepatic ferroptosis, reduce lipid peroxidation and oxidative stress, and protect hepatocytes	SIRT1	In vivo	[136]
	Atractylodin	C_13_H_10_O	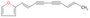	Enhances antioxidant capacity through the SIRT3/PRDX3 pathway, suppressing ferroptosis‐associated lipid peroxidation and protecting the gastrointestinal tract	SIRT3/PRDX3	In vivo	[137]
	5β‐Hydroxycostic acid	C_15_H_22_O_3_	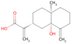	Inhibits NF‐κB and MAPK signaling, reduces inflammation and ferroptosis‐associated lipid peroxidation, and alleviates sepsis‐induced renal injury	NF‐κB, MAPK	In vivo	[138]
	Puerarin	C_21_H_20_O_9_	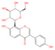	Activates AMPK signaling to suppress ferroptosis‐related lipid peroxidation and reduce cardiomyocyte death in sepsis	AMPK	In vivo	[139]
Autophagy	Monotropein	C_16_H_22_O_11_	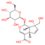	Induces autophagy and alleviates sepsis‐induced colonic injury	NRF2/PINK	In vivo	[141]
	Polydatin	C_20_H_22_O_8_	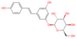	Promotes SIRT6‐dependent autophagy, reducing septic myocardial damage	SIRT6	In vivo	[142]
	Salidroside	C_14_H_20_O_7_	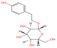	Suppresses NLRP3 inflammasome activation and enhances autophagy, protecting endothelial cells from LPS‐induced injury	NLRP3	In vitro + in vivo	[143]
	Sodium tanshinone IIA sulfonate	C_19_H_17_NaO_6_S	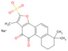	Enhances autophagy and inhibits the NLRP3 inflammasome, preventing endotoxin‐induced cardiomyopathy	NLRP3	In vivo	[144]
	Andrographolide	C_20_H_30_O_5_	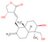	Promotes autophagy in alveolar macrophages through the RAGE/PI3K/AKT/mTOR pathway, improving sepsis‐induced ALI	RAGE/PI3K/AKT/mTOR	In vivo	[145]
	Anemoside B4	C_59_H_96_O_26_	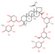	Regulates mTOR/p70S6K‐mediated autophagy and protects against sepsis‐induced acute liver injury	mTOR/p70S6K	In vivo	[146]
NETosis	Zingerone	C_11_H_14_O_3_	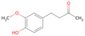	Activates Nrf2, suppresses ROS production, reduces NETs formation, alleviates organ injury, and improves survival	Nrf2/ROS	In vivo	[147]
	Forsythoside B	C_34_H_44_O_19_	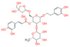	Inhibits PAD4‐dependent NETs formation, improves coagulopathy, and attenuates multiorgan injury	PAD4	In vivo	[148]
	Glycyrrhizin	C_42_H_62_O_16_	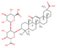	Suppresses the HMGB1/TLR9/MyD88 pathway, reduces NETs formation, improves ARDS, and mitigates lung injury	HMGB1/TLR9/MyD88	In vivo	[149]
	Emodin	C_15_H_10_O_5_	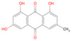	Targets neutrophil BCL‐10 to inhibit NETosis and alleviate sepsis‐induced multiorgan injury	BCL‐10	In vivo	[150]
	Hesperetin	C_16_H_14_O_6_	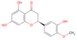	Inhibits ROS and autophagy‐related signaling, reduces NETs formation, suppresses inflammation, and protects intestinal barrier function	ROS/autophagy\ signaling	In vitro + in vivo	[151]
	Itaconate	C_5_H_6_O_4_	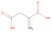	Inhibits HIF‐1α and activates HO‐1, reduces NETs formation, suppresses NETosis, and exerts anti‐inflammatory effects	HIF‐1α/HO‐1	In vitro	[152]
	Resveratrol	C_14_H_12_O_3_	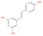	Exhibits antioxidant and anti‐inflammatory effects; suppresses NETs formation by reducing DNA and MPO release	DNA/MPO release	In vitro	[153]
	Catechin	C_15_H_14_O_6_	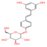				
PANoptosis	Ursodeoxycholic acid	C_24_H_40_O_4_	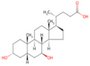	Blocks PANoptosis by inhibiting the STING signaling pathway, significantly reducing sepsis‐induced lung injury	STING	In vivo	[156]
	Sulforaphane	C_6_H_11_NOS_2_	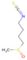	Suppresses LPS‐induced macrophage PANoptosis through inhibition of the TLR4/NF‐κB pathway	TLR4/NFκB	In vitro + in vivo	[157]
	Itaconic acid	C_5_H_6_O_4_	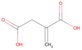	Alleviates macrophage PANoptosis and sepsis‐induced lung injury by inhibiting Ninjurin‐1‐mediated plasma membrane rupture	Ninjurin‐1	In vitro + in vivo	[158]
Necroptosis	Resveratrol	C_14_H_12_O_3_	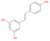	Inhibits the inflammatory response and reduces the expression level of p‐MLKL	p‐MLKL	In vitro + in vivo	[162]

### 5.1 Natural Compounds Regulating Apoptosis

Apoptosis plays a dual role in the onset and progression of sepsis and is a major PCD modality contributing to immune dysfunction and multiple organ failure. On one hand, controlled apoptosis can eliminate overly activated inflammatory cells, such as neutrophils and macrophages, facilitating the resolution of inflammation and supporting tissue repair during the recovery phase of sepsis [105]. On the other hand, excessive apoptosis drives the immune system into a state of profound immunosuppression, increasing susceptibility to secondary infections and contributing to higher mortality [6].

Recent studies indicate that various natural compounds exert protective effects in sepsis by modulating apoptotic pathways. Resveratrol attenuates oxidative stress and inflammation while inhibiting apoptosis through activation of the Sirt1/AMPK/Nrf2 signaling axis [106]. Curcumin suppresses NF‐κB and MAPK signaling, thereby reducing inflammatory cytokine release and limiting sepsis‐associated apoptosis [107, 108]. Paeoniflorin mitigates oxidative stress and inhibits apoptosis via the modulation of the Nrf2/HO‐1 pathway [109]. Quercetin protects against sepsis‐induced organ injury by inhibiting the PI3K/Akt pathway and suppressing apoptosis [110]. Chlorogenic acid activates the Nrf2/HO‐1 pathway, reducing oxidative injury and apoptosis in sepsis models [111]. Ginsenosides also modulate the PI3K/Akt axis to inhibit apoptosis and improve organ dysfunction [112]. Tanshinone exerts anti‐inflammatory and antiapoptotic effects by suppressing the NF‐κB and MAPK signaling [113]. Allicin attenuates oxidative stress and apoptosis via the activation of the Nrf2/HO‐1 axis [114]. Celastrol attenuates LPS‐induced acute hepatic injury by suppressing inflammation, apoptosis, and oxidative stress through MAPK‐ and PI3K/Akt‐related signaling [115]. Byakangelicin displays potent anti‐inflammatory activity by suppressing NF‐κB activation, decreasing the production of IL‐6, TNF‐α, and IFN‐γ, and downregulating apoptosis‐related genes; further studies suggest that these effects may be mediated through modulation of the 26S proteasome regulatory subunit PSMC5 [116].

Most studies on apoptosis‐regulating natural compounds in sepsis rely on LPS‐induced cell or animal models, which do not fully reflect the polymicrobial and dynamic nature of clinical sepsis (e.g., cecal ligation and puncture model is rarely used). The claimed antiapoptotic effects are often inferred solely from changes in Bcl‐2/Bax ratio and Caspase‐3 cleavage by Western blot, without direct quantification of apoptotic cells via flow cytometry or TUNEL staining. Moreover, genetic controls (e.g., Caspase‐3^–/–^ or Nrf2^–/–^ mice) are almost never employed to confirm target specificity. Pharmacokinetic issues—particularly poor bioavailability of compounds like resveratrol and curcumin—are frequently acknowledged but rarely addressed experimentally (e.g., by measuring plasma concentrations or using drug delivery systems). Only a handful of compounds have been tested in large animal models or clinical settings, leaving a wide translational gap.

A summary of natural compounds associated with apoptosis regulation is presented in Table 4. Collectively, these compounds act through multitarget and multipathway mechanisms, demonstrating significant protective effects by attenuating inflammation, suppressing apoptosis, and improving organ dysfunction in sepsis. Although challenges such as low bioavailability and the lack of large‐scale clinical trials hinder their translational application, apoptosis‐targeting natural compounds represent a promising direction for the development of immunomodulatory therapies in sepsis.

### 5.2 Natural Compounds Regulating Pyroptosis

Pyroptosis is a GSDMD‐mediated inflammatory form of PCD that plays a dual role in the pathogenesis of sepsis. While moderate pyroptotic activation contributes to pathogen clearance, its excessive induction promotes cytokine storm, immune dysregulation, and multiorgan dysfunction. Inhibition of NLRP3 inflammasome activation or GSDMD cleavage can alleviate organ injury in sepsis models [117, 118, 119]. In recent years, accumulating evidence has demonstrated that various natural compounds exert antisepsis effects by modulating pyroptosis through multiple targets and pathways. Their principal mechanisms include suppression of NLRP3 inflammasome assembly, blockade of GSDMD processing, attenuation of oxidative stress, and regulation of upstream signaling cascades.

Recent studies have documented that numerous natural compounds exert protective effects in sepsis by modulating pyroptotic pathways. Chitosan oligosaccharides alleviate macrophage pyroptosis in septic mice by activating the Nrf2/GPX4 axis, thereby reducing oxidative stress and inflammation and protecting tissues from sepsis‐induced injury [120]. Corilagin suppresses LPS‐triggered pyroptosis in macrophages by interfering with MyD88 and ASC, thereby impairing NLRP3 inflammasome assembly and limiting inflammatory cytokine release [121]. Americanin B directly binds to NLRP3 in a mouse model of LPS‐induced septic encephalopathy, suppresses pyroptosis, decreases IL‐1β and IL‐18 secretion, and mitigates neuroinflammation and brain injury [122]. Sinapic acid appears to inhibit the activation of the NLRP3 inflammasome by suppressing the NLRP3–ASC–Caspase‐1 signaling cascade, thereby restraining pyroptosis and inflammation [123]. Similarly, dihydromyricetin and phillyrin ameliorate sepsis‐associated ALI by suppressing NLRP3‐dependent pyroptosis and reducing pulmonary inflammation and tissue damage via the Caspase‐1/GSDMD pathway [124, 125]. Taken together, pyroptosis represents a central mechanism driving uncontrolled inflammation and organ injury in sepsis. Natural compounds that modulate pyroptosis via multiple molecular targets offer promising therapeutic potential, providing both conceptual and experimental support for developing novel antisepsis strategies. A summary of natural compounds regulating pyroptosis in sepsis is presented in Table 4.

Although many natural compounds are reported to inhibit NLRP3 inflammasome activation and GSDMD cleavage, the evidence is predominantly based on LPS‐primed macrophages with ATP or nigericin as a second trigger, which does not replicate the complex inflammatory milieu of sepsis. Crucially, few studies directly visualize GSDMD pore formation or measure IL‐1β/IL‐18 release in vivo. The use of selective inhibitors (e.g., MCC950 for NLRP3) or knockout mice (e.g., Gsdmd^–/–^, Casp1^–/–^) for mechanistic validation is rare. Furthermore, most compounds are tested in acute LPS challenge models rather than the more clinically relevant CLP model, and the timing of administration (often prophylactic) limits translational relevance. Without head‐to‑head comparisons, it remains unclear which compound is most effective at inhibiting pyroptosis without compromising host defense against pathogens.

### 5.3 Natural Compounds Regulating Ferroptosis

Ferroptosis is a lipid peroxidation‐driven, iron‐reliant mode of PCD that contributes significantly to multiorgan damage during sepsis. Its core mechanisms include dysregulated iron metabolism, reduced activity of GPX4, accumulation of lipid peroxides, and mitochondrial dysfunction, ultimately leading to membrane damage and cell death. Ferritinophagy‐mediated release of Fe^2^^+^ can further enhance mitochondrial ROS production and exacerbate tissue injury during sepsis [126, 127]. At the molecular level, several pathways—including Keap1–Nrf2–GPX4, p53, and the SLC7A11/xCT antiporter—participate in the regulation of ferroptosis. Compared with other forms of PCD (e.g., apoptosis, necroptosis, pyroptosis, and NETosis), ferroptosis exhibits distinct biochemical and morphological features; however, substantial crosstalk exists among these pathways. Iron overload and ROS not only trigger ferroptosis but can also activate apoptosis, necroptosis, and pyroptosis. Likewise, ferroptosis interacts with necroptosis and NETosis, potentially contributing synergistically to the development of organ dysfunction in sepsis [128].

In sepsis models, several natural compounds have demonstrated protective effects across multiple organs by enhancing Nrf2‐mediated antilipid peroxidation defenses [129, 130, 131, 132]. For example, andrographolide suppresses ferroptosis in renal and pulmonary tissues via Nrf2/FSP1 activation or Keap1–Nrf2 modulation, mitigating oxidative injury and supporting organ function [129, 131]. Flavonoids such as hyperoside, baicalein, and chrysin‐loaded PLGA nanoparticles protect the lung or myocardium from ferroptosis‐driven injury through Nrf2 activation or microRNA‐mediated regulation [133, 134, 135]. Plant‐derived acids, including isoferulic acid, atractylodin, and 5β‐hydroxy‐costic acid, target ferroptosis‐related oxidative stress via SIRT1/SIRT3 or NF‐κB/MAPK pathways, thereby alleviating hepatic, gastrointestinal, and renal injury [136, 137, 138]. Puerarin also confers cardioprotection through an AMPK‐dependent mechanism [139]. Collectively, these findings highlight ferroptosis as a promising therapeutic target in sepsis. Natural compounds that modulate ferroptotic pathways offer potential for multiorgan protection. A summary of these mechanisms is provided in Table 4.

The emerging role of ferroptosis in sepsis has attracted attention, but current natural compound studies suffer from several methodological shortcomings. Many reports rely exclusively on GPX4 and lipid peroxidation markers (e.g., MDA, 4‐HNE) as readouts, without measuring intracellular Fe^2^^+^ levels, ACSL4 activity, or mitochondrial morphology changes—key features of ferroptosis. Ferroptosis‐specific inhibitors (e.g., Ferrostatin‐1, Liproxstatin‐1) are rarely included as positive controls, making it difficult to attribute observed effects specifically to ferroptosis rather than other forms of oxidative cell death. Additionally, most studies use LPS challenge alone, whereas sepsis‐induced ferroptosis is more prominent in CLP models with longer disease courses. The potential crosstalk between ferroptosis and other PCD pathways (e.g., necroptosis, pyroptosis) is almost never experimentally dissected, leaving a major mechanistic gap.

### 5.4 Natural Compounds Regulating Autophagy‐Related Cell Death

Autophagy plays a dual role in sepsis. It serves not only as a cytoprotective mechanism that enables cells to cope with pathogens and stress‐induced injury, but, when excessively or persistently activated, can also lead to autophagy‐dependent cell death. During the early stages of sepsis or under moderate stress conditions, autophagy is generally protective. As an evolutionarily conserved degradation and recycling process, autophagy clears damaged mitochondria, protein aggregates, and intracellular pathogens, thereby preserving cellular homeostasis, reducing oxidative stress, suppressing excessive inflammatory responses, and preventing apoptosis. Through these mechanisms, autophagy contributes to the protection of vital organs such as the heart, lungs, and kidneys. However, in severe or late‐stage sepsis, or within specific cell populations, autophagy may become excessively activated, surpassing the cell's capacity for repair. This uncontrolled or prolonged activation can lead to the degradation of essential cellular components and ultimately evolve into autophagy‐dependent cell death [23, 140].

Autophagy represents a central regulatory node in the pathogenesis of sepsis and is tightly intertwined with multiple forms of PCD. Through switching between survival‐promoting and death‐inducing signals, autophagy engages in extensive crosstalk with apoptosis, necroptosis, pyroptosis, and ferroptosis, collectively determining the fate and functional integrity of immune cells and parenchymal cells. These interactions ultimately influence organ dysfunction and clinical outcomes in sepsis. Consequently, precise modulation of autophagic activity is considered a promising strategy for the treatment of sepsis and multiple organ dysfunction syndrome (MODS).

Numerous natural products originating from traditional Chinese medicine have been shown to confer protection against sepsis through regulation of autophagy‐related pathways. Monotropein activates the NRF2/PINK1 axis to induce autophagy, thereby alleviating sepsis‐induced colonic injury [141]. Polydatin reduces septic cardiomyopathy by promoting SIRT6‐dependent autophagy [142]. Salidroside protects endothelial cells against LPS‐induced inflammatory injury by suppressing NLRP3 inflammasome activation and enhancing autophagy [143]. Sodium tanshinone IIA sulfonate prevents endotoxin‐induced cardiomyopathy through autophagy activation and inhibition of the NLRP3 inflammasome [144]. Andrographolide enhances autophagy in alveolar macrophages via the RAGE/PI3K/AKT/mTOR pathway, thereby improving sepsis‐induced ALI [145]. Anemoside B4 exerts hepatoprotective effects against sepsis‐induced acute liver injury by regulating mTOR/p70S6K‐mediated autophagy [146]. Collectively, these findings underscore the central role of autophagy in mediating the protective actions of natural compounds against multiorgan injury in sepsis, providing a scientific basis for autophagy‐targeted therapeutic strategies. A summary of these mechanisms is presented in Table 4.

While numerous natural compounds are claimed to “induce protective autophagy” in sepsis, a critical distinction between autophagic flux and autophagosome accumulation is frequently overlooked. Most studies simply measure LC3‐II and p62 levels without using lysosomal inhibitors (e.g., chloroquine, bafilomycin A1), making it impossible to determine whether the observed LC3‐II increase reflects genuine flux enhancement or impaired degradation. Moreover, the functional contribution of autophagy to cell survival versus death is rarely dissected by genetic approaches (e.g., Atg5^–/–^ or Atg7^–/–^ cells). Many compounds are tested only in one cell type (e.g., macrophages) at a single time point, ignoring the dynamic nature of sepsis where autophagy may be beneficial early but detrimental later. Finally, the dose–response relationship and off‐target effects (e.g., mTOR‐independent pathways) are poorly characterized, limiting confidence in target specificity [140].

### 5.5 Natural Compounds Regulating NETosis

In sepsis, NETosis plays a critical role in host defense by releasing DNA, histones, and granular proteins that form web‐like structures capable of trapping and eliminating pathogens, thereby limiting the spread of infection. However, excessive or dysregulated NETs release can intensify systemic inflammation, promote microvascular thrombosis, and contribute to organ injury. Aberrant NETosis is closely associated with coagulopathy and declining organ function in septic patients. NETosis also exhibits extensive crosstalk with other forms of PCD. Overactivation of NETosis can release proinflammatory mediators that amplify inflammasome activation, particularly NLRP3‐mediated pyroptosis, and enhance RIPK1/MLKL‐dependent membrane rupture characteristic of necroptosis. Conversely, inflammatory signals generated by pyroptosis and necroptosis can further induce NETosis, creating a self‐reinforcing loop that aggravates systemic inflammation and multiorgan dysfunction. In contrast, apoptosis, a noninflammatory form of cell death, may be indirectly suppressed or disrupted by excessive NETs, contributing to immune imbalance and disease progression. Accumulating evidence indicates that several natural compounds can modulate NETosis and thereby ameliorate sepsis progression [71, 74].

In the CLP‐induced sepsis model, zingerone markedly reduces NETs formation by suppressing ROS production through Nrf2‐dependent mechanisms, thereby alleviating organ injury and inflammatory responses in mice [147]. Forsythoside B mitigates coagulopathy and multiorgan damage in septic rats by inhibiting PAD4‐dependent NETs formation [148]. Glycyrrhizin attenuates NETosis and improves ARDS by suppressing the HMGB1/TLR9/MyD88 pathway [149]. Emodin reduces sepsis‐induced multiorgan injury by targeting neutrophil BCL‐10 to inhibit NETosis [150]. Both hesperetin and itaconate have been shown to reduce LPS‐induced NETs formation; hesperetin acts by inhibiting ROS generation and autophagy‐related signaling to preserve intestinal barrier function, whereas itaconate and its derivatives suppress NETosis through inhibition of HIF‐1α and activation of HO‐1 [151, 152]. Resveratrol and catechin also significantly suppress NETs formation in vitro, exhibiting notable anti‐inflammatory and antioxidant activities [153]. Collectively, these natural compounds modulate key NETosis‐related signaling pathways and exert anti‐inflammatory and organ‐protective effects in sepsis and related animal models. They provide new potential targets and therapeutic candidates for sepsis management. A summary of these mechanisms is presented in Table 4.

NETosis research in sepsis is hampered by inconsistent definitions and detection methods. Many natural compound studies rely solely on extracellular DNA staining or MPO–DNA complex ELISA, which cannot distinguish NETosis from other forms of neutrophil death or simple DNA release. Live‐cell imaging or quantification of citrullinated histone H3 release is rarely performed. The majority of experiments use phorbol myristate acetate as a NETosis inducer, which does not mimic sepsis‐relevant stimuli (e.g., bacteria, LPS, or activated platelets). Importantly, the potential dual role of NETosis—beneficial for pathogen trapping but harmful for microvascular thrombosis—is seldom evaluated in the same model. Few studies assess NETosis in vivo using PAD4^–/+^ mice or DNase I treatment to confirm causality between NET inhibition and improved outcomes. Without such rigorous validation, the translational potential of NETosis‐targeting natural compounds remains uncertain.

### 5.6 Natural Compounds Regulating PANoptosis

As a recently proposed concept describing the coordinated execution of multiple PCD pathways, PANoptosis has gained increasing attention in infectious and inflammatory disease models. Research on PANoptosis in sepsis remains in its early stages, yet emerging evidence indicates that it represents a crucial mechanism contributing to systemic inflammation and multiorgan dysfunction. In sepsis, PANoptosis is triggered through the activation of the PANoptosome complex, which integrates key molecular components such as ZBP1, Caspase‐1/8, and RIPK1/3, ultimately driving cell death and amplifying inflammatory injury. PANoptosis exhibits extensive overlap with apoptosis, necroptosis, and pyroptosis, reflecting shared signaling machinery: for example, both apoptosis and PANoptosis rely on caspase activation; necroptosis and PANoptosis utilize RIPK‐dependent signaling; and pyroptosis shares inflammasome‐related pathways with PANoptosis. These interconnected regulatory networks underscore the integrated nature of PCD pathways in sepsis and highlight that PANoptosis may serve as a central convergence point, exacerbating disease progression [154, 155].

Recent studies have shown that natural compounds and endogenous metabolites can mitigate sepsis progression by modulating PANoptosis pathways. Ursodeoxycholic acid inhibits the STING signaling pathway to block PANoptosis, significantly reducing sepsis‐induced lung injury [156]. Sulforaphane attenuates LPS‐induced PANoptosis in macrophages by suppressing TLR4/NF‐κB signaling, thereby reducing sepsis‐associated ALI [157]. Itaconic acid similarly limits macrophage PANoptosis and protects against sepsis‐induced pulmonary damage through inhibition of Ninjurin‐1‐mediated plasma membrane rupture [158]. Collectively, these findings support the therapeutic relevance of natural compounds that modulate PANoptosis in the context of sepsis. A summary of these mechanisms is presented in Table 4.

PANoptosis is a recently proposed concept, and studies on natural compound regulation in sepsis are still nascent. The main limitation is that most evidence comes from only two or three compounds (e.g., ursodeoxycholic acid, sulforaphane) in macrophage cell lines, with limited in vivo confirmation using PANoptosome component knockout mice (e.g., Zbp1^–/–^, Ripk3^–/–^, Casp1/8^–/–^). Current readouts often measure individual pathways (pyroptosis, apoptosis, necroptosis) separately rather than demonstrating simultaneous activation within the same cell. Moreover, the specific contribution of PANoptosis versus the sum of its individual components is rarely dissected by pharmacological inhibition of single pathways. Whether natural compounds directly disrupt PANoptosome assembly or act on upstream shared signals (e.g., ROS, NF‐κB) remains unclear. Future studies should prioritize codetection methods (e.g., multicolor flow cytometry, single‑cell imaging) and genetic epistasis experiments to establish a causal role for PANoptosis in sepsis.

### 5.7 Natural Compounds Targeting Necroptosis in Sepsis

Beyond its canonical role in necroptotic execution, RIPK3 has context‐dependent, kinase‐dependent, and kinase‐independent functions involving mitochondrial metabolism, inflammasome regulation, autophagy, and immune signaling [159]. Canonical necroptosis is mediated primarily through the RIPK1–RIPK3–MLKL signaling axis, in which MLKL phosphorylation and oligomerization lead to plasma‐membrane permeabilization and the release of DAMPs [160]. In sepsis, RIPK3‐dependent tissue injury has been demonstrated in polymicrobial models, supporting necroptosis‐related signaling as a therapeutically relevant pathway [161].

Despite its mechanistic significance, natural‐compound‐mediated regulation of necroptosis remains insufficiently investigated in sepsis. Resveratrol is among the few natural compounds with experimentally demonstrated antinecroptotic activity in a sepsis‐relevant setting. In a Vibrio vulnificus‐induced sepsis mouse model, resveratrol pretreatment reduced RIPK1/RIPK3 signaling and MLKL phosphorylation in peritoneal macrophages and multiple tissues, including the lung [162]. For most other natural compounds with reported cytoprotective or anti‐inflammatory effects in sepsis, the specific contribution of necroptosis inhibition remains unconfirmed.

Future studies should prioritize mechanism‐driven screening and in vivo validation using necroptosis‐specific endpoints such as p‐RIPK3 and p‐MLKL, together with appropriate pharmacological or genetic controls. Such approaches are required to distinguish genuine necroptosis modulation from nonspecific anti‐inflammatory or cytoprotective effects [159, 160, 161, 162].

### 5.8 Natural Compounds Regulating Cuproptosis in Sepsis

Cuproptosis is a recently identified copper‐dependent form of cell death driven by excess copper binding to lipoylated mitochondrial TCA cycle proteins (e.g., DLAT), leading to protein aggregation, Fe–S cluster loss, and proteotoxic stress [163]. Copper homeostasis is disrupted in sepsis: serum copper levels fluctuate, and ceruloplasmin (a copper‐carrying protein) is an acute‐phase reactant. However, the direct role of cuproptosis in sepsis pathophysiology remains largely unexplored.

As of 2026, no natural compound has been reported to directly modulate cuproptosis in any sepsis model. A few compounds have been studied for copper chelation or copper homeostasis regulation in other diseases (e.g., tetrathiomolybdate in Wilson's disease, curcumin as a copper chelator in cancer models), but none have been tested in sepsis with cuproptosis‐specific endpoints (e.g., DLAT oligomerization, FDX1 expression, or copper retention in mitochondria).

The complete absence of cuproptosis‐focused natural compound research in sepsis represents both a major gap and a high‐impact opportunity. Future investigations should: ① First establish whether cuproptosis occurs in septic tissues (e.g., liver, kidney, heart) by measuring lipoylated protein aggregation, FDX1 levels, and mitochondrial copper content; ② screen natural products with copper‐chelating or copper‐delivering properties (e.g., flavonoids, polyphenols, curcuminoids) in cuproptosis‐sensitive cell lines (e.g., with elesclomol + CuCl_2_ stimulation); ③ test positive hits in CLP models with cuproptosis‐specific readouts and copper chelators (e.g., tetrathiomolybdate) as controls. These substantial evidence gaps indicate that the role of natural‐compound‐mediated cuproptosis regulation in sepsis remains hypothetical and requires direct experimental validation.

### 5.9 Crosstalk Among PCD Pathways and Multitarget Regulation by Natural Compounds

PCD pathways in sepsis do not operate in isolation; instead, they form an interactive regulatory network through shared signaling hubs, including NF‐κB, ROS, and mitochondrial dysfunction [164, 165, 166, 167]. Activation of NF‐κB simultaneously primes NLRP3 inflammasome assembly during pyroptosis, regulates Bcl‐2 family proteins involved in apoptosis, and induces RIPK1 signaling associated with necroptosis [168]. ROS further function as common executors across ferroptosis through lipid peroxidation, pyroptosis via NLRP3 activation, necroptosis through RIPK1–RIPK3 necrosome formation, and NETosis via PAD4‐mediated chromatin decondensation [20, 168]. Mitochondrial damage also contributes to multiple forms of PCD by releasing cytochrome *c*, mtDNA, and excessive ROS, thereby linking mitochondrial quality control to inflammatory and death‐related signaling cascades [169, 170, 171].

The interplay among PCD pathways has important therapeutic implications in sepsis. For instance, inhibition of Caspase‐8 can shift cellular fate from apoptosis toward necroptosis, illustrating the redundancy and compensatory nature of death signaling pathways [168]. Ferritinophagy, a selective form of autophagy targeting ferritin, releases intracellular iron and promotes ferroptosis, whereas autophagy can also eliminate damaged mitochondria to suppress ferroptotic injury [170, 172]. NETosis additionally contributes to pyroptotic signaling in neighboring cells through extracellular histones and proteases [173, 174]. These findings suggest that multitarget therapeutic strategies may provide greater efficacy than interventions directed at a single PCD pathway [175].

Natural compounds are particularly suitable for multitarget regulation because of their ability to modulate shared signaling nodes involved in inflammation, oxidative stress, and PCD. Curcumin suppresses NF‐κB and NLRP3 inflammasome activation while enhancing Nrf2 signaling, thereby attenuating pyroptosis, apoptosis, and ferroptosis [108]. Resveratrol improves sepsis progression by inhibiting excessive autophagy and interfering with NETosis [176, 177]. Andrographolide suppresses ferroptosis through modulation of the Keap1/Nrf2 pathway and promotes autophagy via the RAGE/PI3K/AKT/mTOR axis [129, 131, 145]. Ginsenoside Rg1 activates Nrf2‐related antioxidant signaling pathways, thereby attenuating oxidative stress‐associated ferroptosis, while also promoting autophagy/autophagic activity in disease models [132, 178]. Sulforaphane, a classical Nrf2 activator, suppresses macrophage PANoptosis through inhibition of the TLR4/NF‐κB pathway [157]. Collectively, these findings support the concept that natural compounds may exert therapeutic benefits in sepsis by coordinately targeting multiple interconnected PCD pathways. Preclinical natural compounds targeting specific PCD pathways involved in sepsis are illustrated in Figure 3.

**FIGURE 3:**
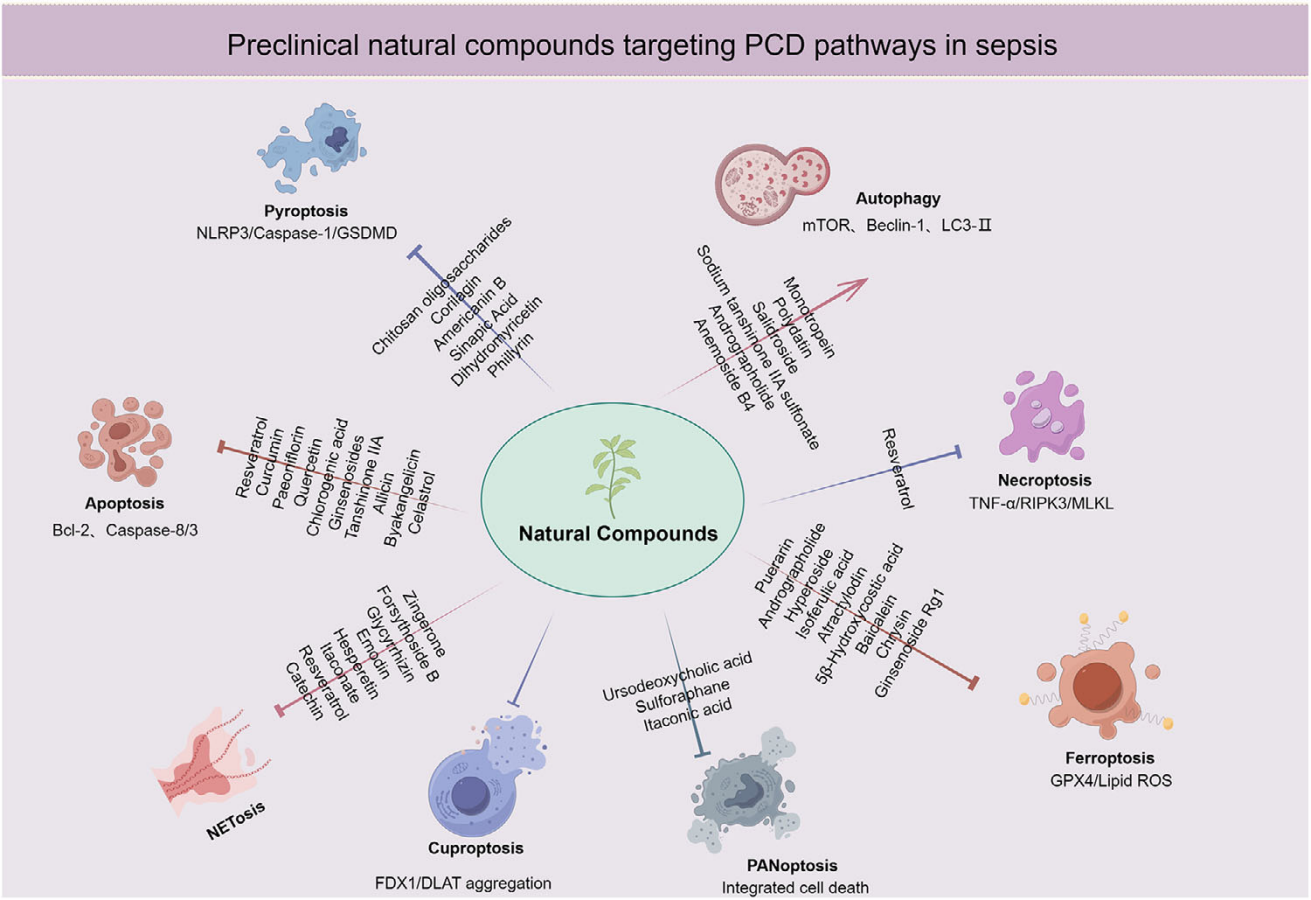
Preclinical natural compounds targeting PCD pathways in sepsis: ① apoptosis: resveratrol; curcumin; paeoniflorin; quercetin; chlorogenic acid; ginsenosides; tanshinone IIA; allicin; byakangelicin; celastrol. ② Pyroptosis: chitosan oligosaccharides; corilagin; Americanin B; sinapic acid; dihydromyricetin; phillyrin. ③ Ferroptosis: puerarin; andrographolide; hyperoside; isoferulic acid; atractylodin; 5β‐hydroxycostic acid; baicalein; chrysin; ginsenoside Rg1. ④ Autophagy: monotropein; polydatin; salidroside; sodium tanshinone IIA sulfonate; andrographolide; anemoside B4. ⑤ NETosis: zingerone; forsythoside B; glycyrrhizin; emodin; hesperetin; itaconate; resveratrol; catechin. ⑥ PANoptosis: ursodeoxycholic acid; sulforaphane; itaconic acid. ⑦ Necroptosis: resveratrol. ⑧ Cuproptosis.

## 6 Discussion

Sepsis remains a major global health burden despite substantial advances in recognition, antimicrobial therapy, hemodynamic management, and organ support. Contemporary evidence supports sepsis as a heterogeneous syndrome in which inflammatory and immunosuppressive programs may coexist and evolve over time [179]. Experimental studies further indicate that integrated programmed cell‐death programs, including PANoptosis, can contribute to sepsis‐associated organ injury [180]. Within this broader network, PCD should be regarded as an important mechanistic layer rather than an isolated disease process.

PCD pathways are interconnected through shared regulators, including NF‐κB, ROS, mitochondrial injury, and caspase/RIPK signaling, creating both therapeutic opportunities and mechanistic complexity [181]. Sepsis‐associated muscle wasting and ICU‐acquired weakness contribute to prolonged functional impairment; apoptosis, mitochondrial dysfunction, proteolysis, and dysregulated autophagy have been implicated, whereas direct evidence that natural compounds improve clinically meaningful muscle outcomes remains insufficient [182].

Guideline‐concordant infection control, hemodynamic stabilization, and organ support remain the foundation of sepsis treatment. Recent randomized trials have primarily refined the delivery and selection of established interventions rather than identified a universally effective new therapy. BLING III, CLOVERS, and CLASSIC illustrate the importance of treatment context in antimicrobial and fluid strategies, whereas ImmunoSep provides proof of concept that immune phenotype may help identify patients more likely to benefit from host‐directed therapy. However, the absence of a demonstrated mortality benefit in ImmunoSep emphasizes that improvement in biomarker‐guided organ‐dysfunction endpoints should not yet be equated with definitive survival benefit.

Experimental natural compounds may modulate multiple PCD‐related pathways, but their apparent pleiotropic effects should not be interpreted as an established clinical advantage. Recent sepsis‐focused literature supports continued investigation of PCD‐directed and natural‐product strategies while emphasizing the need for rigorous mechanistic and translational validation [183, 184].

There exists a significant translational gap between preclinical discovery and clinical application. A critical barrier impeding the clinical translation of natural compounds for sepsis lies in the persistent disconnect between reductionist experimental systems, animal disease paradigms, and the complex pathophysiology of human sepsis [185, 186]. In vitro models routinely employ immortalized cell lines stimulated with LPS at supraphysiological concentrations, typically 1–10 µg/mL. Although such systems facilitate mechanistic dissection, they inherently fail to recapitulate hallmark features of clinical sepsis, including multicellular coordination, spatiotemporally regulated immune cell infiltration, and dynamic interorgan crosstalk. Moreover, concentrations of natural compounds used in vitro may substantially exceed therapeutically attainable systemic exposures because of limited absorption, extensive first‐pass metabolism, and rapid systemic clearance. This pharmacokinetic mismatch may reduce the physiological relevance of some in vitro findings [187, 188, 189, 190].

Animal models also exhibit marked heterogeneity in clinical fidelity. The LPS challenge model offers experimental tractability and reproducibility, but it induces a monophasic, cytokine‐driven hyperinflammatory state that poorly mirrors the polymicrobial etiology, temporal evolution, and biphasic immune trajectory of human sepsis, which is characterized by early hyperinflammation followed by compensatory immunosuppression. In contrast, the cecal ligation and puncture model more faithfully reproduces key clinical dimensions, including microbial diversity, progressive organ dysfunction, and immunometabolic reprogramming [191, 192], yet it suffers from substantial interlaboratory variability in surgical technique, puncture size, resuscitation protocols, and outcome definitions. Alternative models, such as intravenous bacterial infusion and colon ascendens stent peritonitis, remain underutilized due to limited validation or poor scalability. Critically, most preclinical studies administer natural compounds either prophylactically or within minutes of sepsis induction a paradigm starkly misaligned with clinical reality, where patients typically present for care several hours after symptom onset. Such temporal misalignment introduces profound bias, leading to inflated estimates of therapeutic benefit [193].

Clinical translatability remains profoundly limited. Curcumin has been evaluated in a randomized clinical trial in critically ill patients with sepsis [108], whereas the evidence for most other natural compounds discussed in this review remains predominantly preclinical. None has yet been established as standard therapy for sepsis, and none have received regulatory approval for sepsis treatment. Principal translational bottlenecks include inadequate aqueous solubility and low oral bioavailability; the absence of standardized, chemically characterized extracts with insufficient batch‐to‐batch quality control; a paucity of safety and tolerability data in critically ill populations with multiorgan failure; and the lack of validated, mechanism‐informed pharmacodynamic biomarkers to guide rational dose selection and therapeutic monitoring.

To meaningfully bridge these preclinical–clinical divides, future research must adopt a rigorously translational framework. Clinically contextualized animal models, such as cecal ligation and puncture incorporating delayed, guideline‐concordant antimicrobial therapy and hemodynamically targeted fluid resuscitation, should be implemented. Quantitative pharmacokinetic–pharmacodynamic modeling is needed to ensure that in vivo dosing regimens yield systemic and tissue exposures consistent with those achievable and safe in humans. Independent validation in large‐animal models, such as porcine sepsis, should be mandated prior to first‐in‐human studies. Drug‐likeness assessment including solubility, metabolic stability, and membrane permeability must be embedded at the earliest stages of compound prioritization, while advanced delivery platforms such as polymeric nanoparticles and lipid‐based nanocarriers should be concurrently explored to overcome bioavailability limitations. Without such deliberate, evidence‐based refinements to the translational pipeline, even the most biologically compelling natural compounds will continue to face insurmountable barriers to clinical adoption.

Despite substantial progress, most natural compounds remain at the preclinical stage, and their translation is limited by incomplete efficacy and safety evaluation, reliance on acute single‐organ models, and insufficient clinical validation [181, 194, 195]. Poor aqueous solubility, low bioavailability, and limited tissue targeting represent additional barriers. Advanced delivery systems and structural optimization may improve pharmacokinetic properties, tissue exposure, and target selectivity [196, 197, 198]. Therefore, rigorous pharmacokinetic, safety, and efficacy evaluation remains essential before these candidates can advance toward clinical application. To promote the development of this field, several specific research directions have been proposed. CRISPR–Cas9 screening can systematically identify the direct targets of natural compounds in the regulation of PCD, with resistance or sensitivity phenotypes as readout indicators. Establishing a multi‐index evaluation system for PCD, combining flow cytometry to detect markers of apoptosis, pyroptosis, necroptosis, and ferroptosis, will enable high‐throughput comparison of multitarget effects. Structure–activity relationship studies are necessary for optimizing lead compounds (such as curcumin, resveratrol) to improve bioavailability and target selectivity. Time‐specific intervention in the CLP model, using delayed treatment regimens, will better simulate clinical sepsis. Multiomics integration (transcriptomics, proteomics, metabolomics) can identify predictive biomarkers for patient stratification. These feasible approaches will accelerate clinical translation. Finally, although PCD pathways exhibit extensive crosstalk, most existing studies focus on a single mode of cell death and lack systematic analyses of how natural compounds coordinately modulate multiple PCD pathways. The integrative mechanisms summarized in this review [199, 200, 201], therefore, remain incomplete. Future investigations should integrate multiomics approaches, including genomics, transcriptomics, proteomics, metabolomics, and microbiome analyses, to comprehensively delineate the regulatory networks through which natural compounds modulate PCD in sepsis, identify novel biomarkers and therapeutic targets [202], and ultimately advance precision medicine. Future progress in sepsis will likely depend on better integration of clinical phenotyping, mechanistic biomarkers, and treatment timing rather than on a single universally effective host‐directed intervention. PCD‐targeting natural compounds should currently be regarded as experimental candidates whose translational value requires reproducible target engagement, clinically achievable exposure, appropriate disease models, and prospective human validation. Integrating these mechanistic advances with guideline‐based clinical care and biomarker‐informed patient selection may provide a more realistic path toward individualized sepsis treatment.

## Author Contributions

Guang‐Tao Pan and Yu Deng drafted the initial manuscript. Lin‐Rui Ma and Yu‐Tong Li performed the literature collection and evidence organization. Yu Qian, Yi‐Hua Ma, and Hai‐Wei Rao revised the manuscript. Yi‐Zhun Zhu and Pei‐Yu Yan supervised the study concept and revised the manuscript. All authors have read and approved the final manuscript.

## Funding

This study was funded by the National Natural Science Foundation of China (No. 82405280) and Macau SAR Science and Technology Development Fund (File no. 0003/2025/NRP).

## Ethics Statement

The authors have nothing to report.

## Conflicts of Interest

Author Yi‐Zhun Zhu is an Editorial board member of MedComm. Author Yi‐Zhun Zhu was not involved in the journal's review of or decisions related to this manuscript. The other authors declared no conflicts of interest.

## Data Availability

The authors have nothing to report.
